# Automatic Generation of Density-Fitting Auxiliary Basis Sets for All-Electron Dirac–Kohn–Sham Calculations

**DOI:** 10.1021/acs.jpca.5c02772

**Published:** 2025-07-16

**Authors:** Nicoló Antonini, Enrico Ronca, Loriano Storchi, Leonardo Belpassi

**Affiliations:** † Dipartimento di Farmacia, Università G. d’Annunzio Chieti-Pescara, via dei Vestini, Chieti 66100, Italy; ‡ Dipartimento di Chimica, Biologia e Biotecnologie, 201791 Università degli Studi di Perugia, via Elce di Sotto 8, Perugia 06123, Italy; § 9327 CNR Institute of Chemical Science and Technologies “Giulio Natta” (CNR-SCITEC), via Elce di Sotto 8, Perugia 06123, Italy

## Abstract

In this study, we present a general workflow that enables the automatic generation of auxiliary density basis sets for all elements of the periodic table (from H to Og) to facilitate the general applicability of relativistic Dirac-Kohn–Sham calculations. It is an important tool for the accurate description of relativistic effects, including spin–orbit coupling, in molecules containing heavy elements. The latter are very important in various fields, ranging from catalysis to quantum technologies. The automatic generation algorithm is based on an even-tempered scheme inspired by a previous work by P. Calaminici et al. *J. Chem. Phys.* **2007**, 126, 044108, in which the auxiliary basis sets were generated for nonrelativistic DFT calculations within the GGA approximation. Here, the algorithm uses basic information from the principal relativistic spinor basis set (exponents and angular momentum values) and includes a simple strategy to account for the high angular momentum of electrons in heavy and superheavy elements. The workflow developed here allows us to perform extensive automated tests aimed at verifying the accuracy of the auxiliary basis sets in a large molecular data set of about 300 molecules representing all groups and periods of the periodic table. The results show that our auxiliary basis sets achieve high accuracy, with errors in the Coulomb energies of a few μ-hartree, which are of the same order of magnitude as in the nonrelativistic density fitting. The automatic workflow developed here is general and will be applied in the future for the optimization of auxiliary basis sets to include exact exchange in relativistic approaches. The latter will be a crucial step for the accurate description of spectroscopic properties and spin dynamics in molecular systems containing heavy elements.

## Graphical abstract

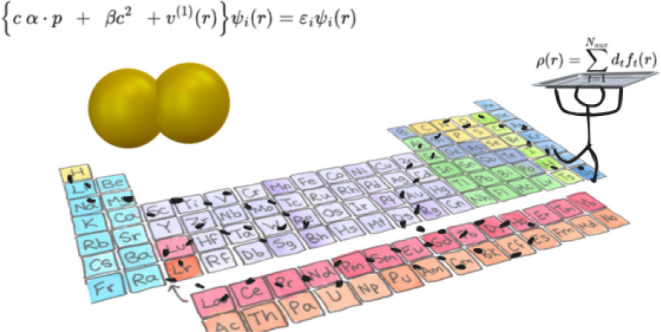

## 1 Introduction

Heavy elements are of great interest in various fields, including catalysis, optoelectronic, spintronics and other quantum technologies. Accurate simulations of molecular systems containing heavy elements require the correct inclusion of relativistic effects (spin–orbit coupling and scalar relativistic corrections), electron correlation and, for very accurate predictions of spectroscopic constants, also QED corrections.^1,2^ In particular, spin–orbit coupling may influence laser-induced spin dynamics and unexpected phenomena such as chirality-induced spin selectivity.^3^

The most accurate way to introduce relativity in the modeling of molecular systems is to use the four-component (4C) formalism derived from the Dirac equation. The full 4C formalism is particularly interesting since it represents the most rigorous way of explicitly treating all the interactions involving spin, which are today of great technological importance. Furthermore, rigorous relativistic theories offer the natural framework to describe the interaction of particles with electromagnetic fields. As a matter of fact, when determining the electronic structure of molecules containing heavy elements, the effects of electron correlation are just as important as those of the theory of relativity. These effects are generally not additive and should be treated on the same footing. Due to the large number of electrons that have to be correlated in systems containing heavy atoms, explicit wave function-based electron correlation methods rapidly exceed practicability limits. A promising approach to include the electron correlation is given by the relativistic four-component generalization of the Kohn–Sham scheme referred to as the Dirac-Kohn–Sham (4C-DKS) method. The practical application of the 4C-DKS methods was hampered by the high computational effort required to properly evaluate the relativistic electron repulsion integrals and all associated properties. To alleviate these limitations, the implementation of density fitting approach was a tremendous breakthrough in reducing the computational burden. The latter was applied in the 4C framework since 2006 in the 4C molecular code BERTHA.^4^ The idea of incorporating the density expansion obtained from the Coulomb fitting directly into the exchange-correlation functional (in local and semilocal approximation) was also particularly effective in further reducing the cost of DKS calculations.^5^ The introduction of the so-called variational density fitting scheme has proven to be particularly effective in combination with the implementation of various parallelization and memory distribution schemes.^6–9^ Recently, we have shown that the DKS method based on density fitting can be easily ported to GPUs.^10^

The density fitting scheme is now successfully applied in other 4C implementations.^11,12^ The density fitting was also used for Dirac–Fock using the Coulomb, Gaunt, and full Breit interactions.^12^ Despite the proven advantages of density fitting, its systematic application in a 4C-relativistic context is still somewhat limited. The density fitting requires a preoptimized atomically centered auxiliary basis set. So far,^4^ we have used an atom-optimized auxiliary basis set that follows a very simple two-step optimization procedure where 1) the exponents optimized on the spherical atom (see Quiney et al.^13^) and 2) angular momentum determined to reduce the error in the Coulomb energy below a required threshold. In refs. 11,12 , for example, the auxiliary basis sets used were specifically generated by an adjusted even-tempered algorithm. In all cases, there was no systematic analysis of the accuracy when the auxiliary basis sets are used in molecules. Typically, only a few molecular systems are used, leaving a significant gap between the benefits of the density fitting technique and its general applicability in routine calculations. Furthermore, while there are many well-established molecular data sets to test the performance of various auxiliary fitting basis sets in nonrelativistic context, they are much more limited for all electron calculations of molecules containing heavy and superheavy elements.

In recent years, there has been increasing interest in the topic of automatically generated Auxiliary Basis Sets (ABS). One of the advantages of these is that they retain the information on the principal basis set and therefore have greater flexibility compared to optimized ABS, which are typically generated using an energetic minimization criterion for the atoms. This is particularly important for applications such as spectroscopic simulations, where the optimized nature of the principal basis set must be retained in the auxiliary set. Among others, we cite the remarkable works by Lehtola,^14,15^ Stoychev,^16^ Köster et al.,^17^ Aquilante et al.,^18–20^ Hellman et al.,^21^ and Yang et al.^22^ that help to understand the actual state of development in this field. Most automated generation strategies rely on decomposition techniques, such as Cholesky decomposition, which allow a minimal but sufficient amount of auxiliary functions to be extracted. Alternatively, one can generate auxiliary functions using some direct information from the principal atomic orbital basis set. The implementation is relatively simple, but one may introduce some uncontrolled errors. In the Cholesky decomposition-based approach, the auxiliary basis set is evaluated on-the-fly, the accuracy is controlled by a single parameter, but an efficient implementation can be nontrivial and the auxiliary basis sets are usually larger than in density fitting. Despite the widespread application in nonrelativistic contexts or even in relativistic two-component,^23^ the generalization to 4C is not straightforward. The difficulty arises from the fact that it is necessary to deal with basis functions that have different properties, including those associated with the large and small components of the spinorial structure of the principal basis set. Recently, Li et al.^24^ proposed a Cholesky integral decomposition based on the Pauli quaternion representation for the relativistic Dirac-Coulomb Hamiltonian and presented an approximated strategy to evaluate the Cholesky vectors for the (SS|LL) block integrals. Unfortunately, the error in the evaluation of these integrals is not bounded by the Cholesky threshold used for the large block. However, no variational collapse is observed in actual numerical tests, suggesting that the Cholesky decomposition may offer promising advantages in terms of memory storage and efficiency.

The accuracy of the density fitting approach, and in particular the automatically generated ABS, can only be assessed on the basis of extensive benchmark calculations on a large number of molecules with atoms in different molecular environments and oxidation states. These benchmarks can be very time-consuming. In general, the molecular data sets are not specific to relativistic calculations and heavy and superheavy elements are under-represented. A notable exception is the molecular data set proposed by Pollak et al.^25^ which includes molecules with atoms up to Rn.

In this study, we have developed a fully automated method for generating relativistic auxiliary basis sets, tested the accuracy and analyzed the results using a large molecular set of about 290 molecules representing all periods and groups of the periodic table (from H to Og). While the details of the algorithm are specific to the structure of the auxiliary basis set (Hermite-Gaussian type functions) used in the DKS module of the BERTHA package,^9,26,27^ the automated workflow is completely general and can be easily adapted to other quantum chemistry codes. In particular, we have automated the computational processes at each stage of the workflow, i.e., from the initial selection of basis sets to the final validation of the results. The automation has also extended to data manipulation and analysis, where we have used Python-based tools for benchmarking and visualization.

The paper is organized as follows. Section 2 gives a brief overview of the main theoretical aspects of the DKS method as implemented in BERTHA, with particular reference to the variational density fitting implementation and to the definition of the auxiliary fitting basis set. Section 3 describes the automatic generation algorithm starting from a given principal G-spinor basis set. In Section 4 we describe the fully automated workflow. In Section 5, instead, we present the overall performance in terms of accuracy, measured as mean error (and standard deviation) on the Coulomb energy, using as reference the DKS calculation without density fitting. Finally, we draw some conclusions and perspectives in Section 6.

## 2 Theoretical Background

In the present section, we will review the basic theory of the 4-components Dirac-Kohn–Sham formalism, with a focus on the aspects specifically related to the G-spinor basis sets and the variational density fitting approach as implemented in the BERTHA code. The 4C-DKS equation (leaving *e*, *m* and ℏ out of the formulation as we are working in atomic units) can be written as

1 $$\left\{c\alpha \cdot p+\beta c^{2}+v^{L}\left(r\right)\right\}\Psi_{i}\left(r\right)=E_{i}\Psi_{i}\left(r\right)$$

where *c* is the speed of light in vacuum, ***p*** is the electron momentum,

$$\alpha =\left(\begin{array}{cc} 0 & \sigma \\ \sigma & 0 \end{array}\right)\;\mathrm{and}\;\beta =\left(\begin{array}{cc} I & 0\\ 0 & -I \end{array}\right)$$ 2

where σ*=(*σ_x_, σ_y_, σ_z_ *)* , with σ_q_ is a 2× 2 Pauli spin matrix and *I* is a 2× 2 identity matrix.

The effective longitudinal potential *v* ^*L*^(**r**) is given by the sum of the scalar external potential generated by the nuclei *v*_ext_[(**r**)], the longitudinal Coulomb interaction $v_{H}^{L}\left[\rho \left(r\right)\right]$ and the exchange-correlation potential $v_{xc}^{L}\left[\rho \left(r\right)\right]$ . The latter is associated with the longitudinal exchange-correlation energy. Its exact form is, like that of the corresponding nonrelativistic quantity, unknown and has to be approximated. Typically, nonrelativistic exchange-correlation functionals are employed. Note that the full Breit term, which contributes to the transverse part of the Hartree interaction, is not considered here.^28,29^ Note that for the large class of time-reversal invariant systems (that is, closed-shell molecules) it does not contribute anyway. In BERTHA, the spinor solution can be expressed as linear combination of G-spinor basis functions ( $M_{\mu }^{\left(T\right)}\left(r\right)$ , *T* = L,S) in the form of

$$\Psi_{i}\left(r\right)=\left[\begin{array}{c} \overset{N}{\underset{\mu }{\sum }}c_{\mu i}^{L}M_{\mu }^{\left(L\right)}\left(r\right)\\ i\overset{N}{\underset{\mu }{\sum }}c_{\mu i}^{S}M_{\mu }^{\left(S\right)}\left(r\right) \end{array}\right]$$ 3

G-spinor are two-component objects derived from spherical Gaussian-type functions. The small component $\left(M_{\mu }^{\left(S\right)}\left(r\right)\right)$ of a G-spinor is defined from the large component $\left(M_{\mu }^{\left(L\right)}\left(r\right)\right)$ via the restricted kinetic balance relation. Details for the G-spinor definitions can be found in refs 29–31 . μ is a collective index that comprises the fine-structure quantum number *k*, the quantum number associated with the *z* component of the angular momentum, *m*_*j*_, the Gaussian exponent, and the origin of the local coordinate system. It is often convenient to label the G-spinors using the nominal orbital angular momentum label, *l*, because this makes the correspondence with nonrelativistic theory more immediate. This is very useful because it allows to build relativistic basis sets starting from a conventional nonrelativistic definition of the basis functions. Among their characteristics, G-spinor basis set does not suffer from the variational problems as the kinetic balance prescription avoids the variational collapse.^32^

In the G-spinor representation of eq 3, the total charge density can be defined as follows

4 $$\rho \left(r\right)=\underset{T=L,S}{\sum }\underset{\mu \nu }{\sum }D_{\mu \nu }^{TT}\rho_{\mu \nu }^{TT}\left(r\right)$$

with *T* = *L*, *S*. The matrices D^*LL*^ and D^*SS*^ are the large- and small-component density matrices

5 $$D_{\mu \nu }^{TT}=\underset{a}{\sum }c_{a\mu }^{T*}c_{a\nu }^{T}$$

where $\left\{c_{a\nu }^{T}\right\}$ is the set of coefficients of the molecular spinor expansion (see eq 3), and the sum over *a* includes only occupied positive energy states (electrons).

Here, we can appreciate a particular aspect of the 4C-DKS scheme implemented in BERTHA. Indeed, because of the specific definition of G-spinor basis we can express the overlap components $\rho_{\mu \nu }^{TT}\left(r\right)=M_{\mu }^{(T)†}\left(r\right)M_{\nu }^{\left(T\right)}\left(r\right)$ as the linear combination of Hermite Gaussian Type Functions (HGTF), according to the following equation:

$$\rho_{\mu \nu }^{TT}\left(r\right)=M_{\mu }^{(T)†}\left(r\right)M_{\nu }^{\left(T\right)}\left(r\right)$$ 6

7 $$=\underset{ijk}{\sum }E_{0}^{TT}\left[\mu ,\nu ;i,j,k\right]H\left[\alpha ,r_{A};i,j,k;r\right]$$

where

8 $$H\left[\alpha ,r_{A};i,j,k;r\right]=\frac{\partial^{i}}{\partial x^{i}}\frac{\partial^{j}}{\partial y^{j}}\frac{\partial^{k}}{\partial z^{k}}e^{-\alpha |r-r_{A}{|}^{2}}$$

The definition and construction of the *E* ^*TT*^_0_ coefficients enables the efficient analytic evaluation of all multicenter G-spinor Coulomb integrals. We recall that the full 4C relativistic code BERTHA is built around an efficient algorithm for the analytical evaluation of relativistic electron repulsion integral, originally developed by Quiney and Grant more than 20 years ago,^29,30,33^ which represents the relativistic generalization of the McMurchie-Davidson algorithm.^33,34^ All the 4-components structure of this formalism is retained into the *E* ^*TT*^_0_ coefficients. Other significant advantages of this approach will emerge in the course of the following discussion in relation to the density fitting implementation.

The matrix representation of the DKS operator in the G-spinor basis is

9 $$H_{\mathrm{DKS}}=\left[\begin{array}{cc} v^{LL}+J^{LL}+K^{LL}+mc^{2}S^{LL} & c\Pi^{LS}\\ c\Pi^{SL} & v^{SS}+J^{SS}+K^{SS}-mc^{2}S^{SS} \end{array}\right]$$

The generalized eigenvalue matrix to be solved is given by

10 $$H_{\mathrm{DKS}}\left[\begin{array}{c} c^{L}\\ c^{S} \end{array}\right]=E\left[\begin{array}{cc} S^{LL} & 0\\ 0 & S^{SS} \end{array}\right]\left[\begin{array}{c} c^{L}\\ c^{S} \end{array}\right]$$

where **c** ^*T*^ are vectors, and v^*TT*^, J^*TT*^, K^*TT*^, S^*TT*^, Π^*TT̅*^ are all matrices. The matrix elements mentioned in the above equation are defined as follows

11 $$S_{\mu ,\nu }^{TT}=\int M_{\mu }^{(T)†}\left(r\right)M_{\nu }^{\left(T\right)}\left(r\right)dr$$

12 $$\Pi_{\mu ,\nu }^{T\mathrm{T̅}}=\int M_{\mu }^{(T)†}\left(r\right)\left(\sigma \cdot p\right)M_{\nu }^{\overset{®}{\left(T\right)}}\left(r\right)dr$$

13 $$v_{\mu ,\nu }^{TT}=\int M_{\mu }^{(T)†}\left(r\right)v_{ext}\left(r\right)M_{\nu }^{\left(T\right)}\left(r\right)dr$$

14 $$J_{\mu ,\nu }^{TT}=\int M_{\mu }^{(T)†}\left(r\right)v_{H}^{L}\left[\rho \left(r\right)\right]M_{\nu }^{\left(T\right)}\left(r\right)dr$$

15 $$K_{\mu ,\nu }^{TT}=\int M_{\mu }^{(T)†}\left(r\right)v_{xc}^{L}\left[\rho \left(r\right)\right]M_{\nu }^{\left(T\right)}\left(r\right)dr$$

where $S_{\mu ,\nu }^{TT}$ , $\Pi_{\mu ,\nu }^{T\mathrm{T̅}}$ and $v_{\mu ,\nu }^{TT}$ are respectively the elements of the overlap matrix, kinetic operator and the external potential *v*_ext_(r) due to the nuclei. The matrix elements $J_{\mu ,\nu }^{TT}$ and $K_{\mu ,\nu }^{TT}$ are associated with the Coulomb operator, $v_{H}^{L}\left[\rho \left(r\right)\right]$ and the exchange-correlation potential, $v_{xc}^{L}\left[\rho \left(r\right)\right]$ , respectively. The matrix *H*_*DKS*_ depends, through ρ in $v_{xc}^{L}\left[\rho \left(r\right)\right]$ and $v_{H}^{L}\left[\rho \left(r\right)\right]$ , on the canonical spinor-orbitals produced by its diagonalization, so that the solution­(c^T^) cannot be obtained in a single step, but in a SCF procedure.

The most expensive steps for a 4C-DKS calculation are the evaluation of the Coulomb and exchange correlation matrix elements (eqs 14 and 15). We have shown that these computational tasks can be reduced by using the density fitting approach,^4,9,29^ Substantially, from a *O*(*N* ^2^) scaling for the total charge density, written in the terms of overlap spinor density (eq 4), one can reduce the scaling factor to linear using auxiliary basis functions (*f*_*t*_(**r**)):

16 $$\overset{∼}{\rho }\left(r\right)=\underset{t}{\sum }d_{t}f_{t}\left(r\right)$$

The density fitting scheme relies on selecting an auxiliary basis set, *f*_*t*_, and determining the coefficients (*d*_*t*_) that best approximate the total electron density. This approach not only simplifies the construction of the required matrices but also avoids the evaluation of four-center in favor of three-center two electron integrals with a minimal loss of accuracy. The coefficients *d*_*t*_ are chosen to minimize the error between the fitted density ρ̃(r) and the true density ρ­(r) using the Coulomb metric $\left(g\left(r-r^{\prime }\right)=\frac{1}{\left|r-r^{\prime }\right|}\right)$ :

17 $$\Delta =\frac{1}{2}\int dr\int dr^{\prime }\left[\rho \left(r\right)-\overset{∼}{\rho }\left(r\right)\right]g\left(r-r^{\prime }\right)\left[\rho \left(r^{\prime }\right)-\overset{∼}{\rho }\left(r^{\prime }\right)\right]$$

This approach is widely used in nonrelativistic cases and has been extensively studied.^35^ The variational Coulomb fitting method, often simply referred to Coulomb fitting, ensures a positive-definite metric, leading to a minimization procedure of Δ, *w*hich is bound from below and gives a linear system for the fitting coefficients **d**:

18 Ad=v

where *A*_*st*_ represents the Coulomb matrix of auxiliary functions:

$$A_{st}={\langle f}_{s}\left|\right|f_{t}\rangle \equiv \int f_{s}\left(r\right)\frac{1}{\left|r-r^{\prime }\right|}f_{t}\left(r^{\prime }\right)drdr^{\prime }$$ 19

The vector v projects the electrostatic Coulomb potential onto the fitting functions:

20 $$v_{s}=\left\langle f_{s}\right|\left|\rho \right\rangle =\underset{\mu \nu }{\sum }\left(I_{s,\mu \nu }^{LL}D_{\mu \nu }^{LL}+I_{s,\mu \nu }^{SS}D_{\mu \nu }^{SS}\right)$$

which, as we can see, can be expressed in terms of the density matrix elements $D_{\mu \nu }^{LL}$ and of the 3-center two-electron repulsion integrals $I_{s,\mu \nu }^{TT}$

21 $$I_{s,\mu \nu }^{TT}=\left\langle f_{s}\right|\left|\rho_{\mu \nu }^{TT}\right\rangle$$

involving both the fitting functions *f*_*s*_ and the charge overlap terms $\rho_{\mu \nu }^{TT}$ .

The Coulomb matrix, therefore, can be expressed as

22 $${\overset{∼}{J}}_{\mu \nu }^{TT}=\left\langle \overset{∼}{\rho }\right|\left|\rho_{\mu \nu }^{TT}\right\rangle =\overset{N_{\mathrm{aux}}}{\underset{t=1}{\sum }}I_{t,\mu \nu }^{TT}d_{t}$$

As soon as the density-fitting approach is used to calculate the Coulomb contribution, the calculation bottleneck shifts to the evaluation of the exchange-correlation matrix. The original idea of incorporating the density expansion obtained from the Coulomb fitting into the exchange-correlation energy functional and potential was introduced by Laikov many years ago.^36^ This original approach was not variational due to the use of mixed densities in the evaluation of the exchange-correlation terms. This limitation was fully recognized and corrected in the references^37^ and.^38^ A comprehensive analysis of Dunlap,^39^ later, confirmed the variational robustness of these schemes, especially in the context of analytical derivative calculations. Of all the effective implementations proposed so far, we have followed the scheme proposed by Köster et al.^37^

At this point, after some derivations (see refs. 29,37 for details), we can obtain the exchange-correlation matrix contribution, similar to eq 18, solving a linear system for a new set of coefficients {*z*}­

23 Az=w

where

24 $$w_{s}=\left\langle v_{xc}\left[\overset{∼}{\rho }\right]\right|f_{s}\rangle$$

The above integral is evaluated numerically.

The total $\left({\overset{∼}{J}}_{\mu \nu }^{TT}+{\overset{∼}{K}}_{\mu \nu }^{TT}\right)$ matrix contribution can be computed in a single step:

25 $${\overset{∼}{J}}_{\mu \nu }^{TT}+{\overset{∼}{K}}_{\mu \nu }^{TT}=\overset{N_{s}}{\underset{t=1}{\sum }}I_{t,\mu \nu }^{TT}\left(d_{t}+z_{t}\right)$$

in terms of three-index two electron integrals $\left(I_{t,\mu \nu }^{TT}\right)$ . The application of such method achieves high-accuracy numerical integration and scales as *N*_*s*_ . *N*_grid_ instead of *N* ^*2*^ . *N*_grid_ . The effectiveness of this approach is further enhanced by using, as fitting functions, primitive HGTF that are grouped together in sets sharing the same exponents. The sets are formed so that to an auxiliary function of given angular momentum are associated all the functions of smaller angular momentum. For instance, a *d* auxiliary function set contains ten primitive Hermite Gaussians, one *s*, three *p*, and six *d* functions all with the same exponent. This scheme allows us to use efficient recurrence relations of Hermite polynomials in the computation of two-electron integrals. Analogous schemes have been adopted in refs. 40–42 .

## 3 Automatic Generation of ABS

The fundamental component of the density fitting scheme is the possibility of having an accurate auxiliary basis set. As already mentioned, there are two main approaches to obtain an ABS: 1) through an optimization procedure based on atomic or molecular calculations; or 2) through an automatic generation procedure starting from a principal basis set. The algorithm described here automatically generates an ABS for a given principal G-spinor basis set. The algorithm presented below does not pretend to be optimal, but as will be shown in the next section, it allows us to generate ABSs that are surprisingly accurate for the entire periodic table. The algorithm is specific for the ABS structure we use in BERTHA, which, as already stated, is formed of primitive HGTFs grouped in sets sharing the same exponents. Based on this definition, only two parameters are required to define each group of HGTFs: the exponent (α) and the associated angular momentum value (*l*_*Fitt*_). The group is formed in such a way that all functions with an angular momentum smaller than *l*_*Fitt*_ (in eq 8 the indexes *i*, *j*, *k* assume all values with the condition that *ij* + *k* ≤ *l*_*Fitt*_) belongs to the auxiliary function with the same exponent, α. The total number of HGTFs for a given exponent, α, are given by

26 $$N_{\alpha }=\frac{\left(l_{\mathrm{fitt}}+3\right)\left(l_{\mathrm{fitt}}+2\right)\left(l_{\mathrm{fitt}}+1\right)}{6}$$

For example, for a value of *l*_fitt_ = 3 we have a total of 20 primitive Hermite-Gaussians, one *s*, three *p*, six *d* and ten *f* functions, all of which have the same exponent (α). The assignment of this angular momentum label is a crucial step in the construction of the basis set. It assumes particular importance for heavy elements which contain electrons with high angular momentum values. An effective algorithm should select value of *l*_fitt_ to achieve an optimal trade-off between computational cost and accuracy.

To generate the ABS exponents, we used an algorithm developed several years ago (see ref.^43^ for the deMon2k code). It was originally used to generate the ABS for elements up to the 3*d* elements (from H to Kr) in nonrelativistic DFT calculations using LDA or GGA exchange-correlation functionals. In this context, the ABSs generated with this algorithm are referred to as GEN-A*n* (see ref.^44^ for details). This algorithm is based on an almost even-tempered method and uses as input the primitive Gaussian exponents of the principal basis set (the smallest β_min_ and the largest, β_max_). The latter values, together with the integer parameter *n*, determine the total number of exponents (*N*_exp_) that must be generated, according to the following formula:

27 $$N_{\exp}=\mathrm{Int}\left(\frac{\ln\left(\beta_{\max}/\beta_{\min}\right)}{\ln\left(6-n\right)}+0.5\right)$$

Here *n* can be assigned an integer value of 2, 3 or 4, which increases the total number of exponents to be generated accordingly.

The tightest seed for the geometric progression (α_0_) is defined as follows:

28 $$\alpha_{0}=2\beta_{\min}{\left(6-n\right)}^{\left(N-1\right)}$$

Then, the first exponent of the fitting set is given by

29 $$\alpha_{1}=\left(1+\frac{n}{12-n}\right)\alpha_{0}$$

and the second one reads as

30 $$\alpha_{2}=\left(\frac{\alpha_{0}}{6-n}\right)$$

Finally, we just need to apply the following geometric progression until all the required *N*_exp_ exponents have been generated

31 $$\alpha_{i+1}=\left(\frac{\alpha_{i}}{6-n}\right)$$

Once we have the set of exponents (corresponding to a certain value of *n*), we need to assign the angular part. This means that we need to set a value of *l*_*Fitt*_ to each exponent. This is an important step in our algorithm as it greatly affects both the accuracy and the computational cost. We use a very simple method here. As already mentioned, G-spinors can be constructed from a conventional, nonrelativistic definition of the basis functions, which are given as terms of Gaussian exponents (β) with respect to the orbital angular momenta *l*. Based on this criterion, we double the values of the exponents for each value of angular momentum and define blocks of exponents in the range from 2β_min,*l*_ to 2β_max,*l*_. This gives us a simple procedure for assigning an angular momentum value to our group of suitable exponents. In particular, if an exponent α_*i*_ of our ABS is 2*β*_min,*l*_≤*α*_*i*_≤2*β*_max,*l*_ , we temporarily assign an angular value (*l*_*temp*_) corresponding to that of the principal basis set (*l*). In those cases where an exponent (α_*i*_) falls into multiple blocks of with different *l* of the principal basis set, we assign to it the largest *l* value. This strategy is useful because it extracts the angular momentum information from the principal basis set, but using these values of *l*_*temp*_ directly in the ABS definition, gives inaccurate results. This is not surprising given the low angular flexibility. Indeed, the exact expansion of two spherical harmonics with angular quantum number *l*_1_ and *l*_2_ involve a linear combination of spherical harmonics of order *L* weighted by Clebsch-Gordan coefficients

32 $$Y_{l_{1}}^{m_{1}}Y_{l_{2}}^{m_{2}}=\overset{l_{1}+l_{2}}{\underset{L=\left|l_{1}-l_{2}\right|}{\sum }}C\left(l_{1},l_{2},L,m_{1},m_{2}\right)Y_{L}^{m_{1}+m_{2}}$$

with *L*-values in the range from |*l*_1_-*l*_2_| to *l*_1_+*l*_2_. This means that in order to fully capture the overlap expansion, combinations of angular values up to *l*_1_+*l*_2_ would have to be considered. Since the small component of the G-spinors is generated via the kinetic equilibrium prescription (which performs a spatial derivative), the expansion would even lead to a higher order spherical harmonic (up to *L*=|*l*_1_+*l*_2_+2|), see ref.^31^). This simple analysis shows that if the generated exponent falls into the *f*-shell (l = 3), we would have to assign *l*_fitt_=*l*_1_+*l*_2_+2=*l*_1_∗2+2=8 . It is easy to see that if large polarized basis sets were used for heavy elements, the number of matching functions would quickly explode. Fortunately, in the Dirac-Kohn–Sham method, which uses local (LDA) or semilocal (GGA) exchange correlation functions, the G-spinor overlap density is always contracted with the one-particle density matrix (see eq 4). This greatly reduces the angular dependence of the quantities involved and it is actually not necessary to include such a strong angular dependence in ABS as eq 32 would suggest. The situation is different if we use density fitting in calculations with exchange-correlation functions that contain a certain amount of exact Hartree–Fock exchange (see refs. 12,17 ). We have pragmatically found that, starting from the *l*_*temp*_ values defined above, it is sufficient to increase them by one or two units to obtain reasonably accurate results. This prompted us to define two different cases

$$l_{\mathrm{fitt}}=l_{\mathrm{temp}}+1;l_{\mathrm{fitt}}=l_{\mathrm{temp}}+2$$ 33

For each of these cases, we have generated two different ABS by setting *n* = 2 and *n* = 3, resulting in four different ABS, namely GEN-n2-v1 (*n* = 2, *l*_*Fitt*_ = l_temp_+1), GEN-n2-v2 (*n* = 2, *l*_fitt_ = *l*_temp_+2), GEN-n3-v1 (*n* = 3, *l*_fitt_ = *l*_temp_+1) and GEN-n3-v2 (*n* = 3, *l*_fitt_ = *l*_temp_+2).

To summarize, to fully generate the ABS we need to retrieve the following information from the main base set:

- the highest (β_max_) and lowest (β_min_) exponents
- the highest and lowest exponents for each atomic shell present in the principal basis (necessary for the assignment of the *l*_temp_ and then the *l*_fitt_ value)

The procedure was implemented in Python and is freely available.^45^

## 4 Computational Details

In the present section we will give some details about the computational procedure we have adopted to efficiently automatize the entire workflow which allowed us to generate different ABSs and efficiently test their accuracy on a large set of molecules.

The workflow is depicted in Figure 1. It begins by selecting a principal G-spinor basis set, which is used to generate the auxiliary basis set (performed by the Python script *fitt_gen*).^45^ The cited Python script can also be used to directly download any desired basis set from the Basis Set Exchange (BSE)^46^ Web site. The algorithm described in the previous section will then automatically generate the auxiliary basis sets for each atom. The generated basis and fitting sets are stored in a JSON file via the berthainputbasis Python modules.^47^ berthainputbasis is a Python module that has been developed to collect and organize all G-spinor basis sets and auxiliary basis sets in a single JSON file. The JSON file, together with the molecular geometry files (in XYZ format), is then used by the berthaingen^48^ module to construct the input file to perform the DKS calculation in BERTHA. While the berthaingen can be used as a stand-alone Python script, it can also be imported as a proper Python module. Subsequently, the *job_submitter* ^45^ Bash script, that automates processing of input generation and calculations submission, has been developed. The *self_tab* ^45^ program, which serves as a CSV database generator, collects all the data and parses them into a single CSV file. Finally, the *data_analysis* Jupyter notebook streamlines statistical analysis by performing data manipulation and visualization. This workflow enhances automation, simplifies the data analysis and facilitates auxiliary function development across a large set of molecular structures.

**Figure 1:**
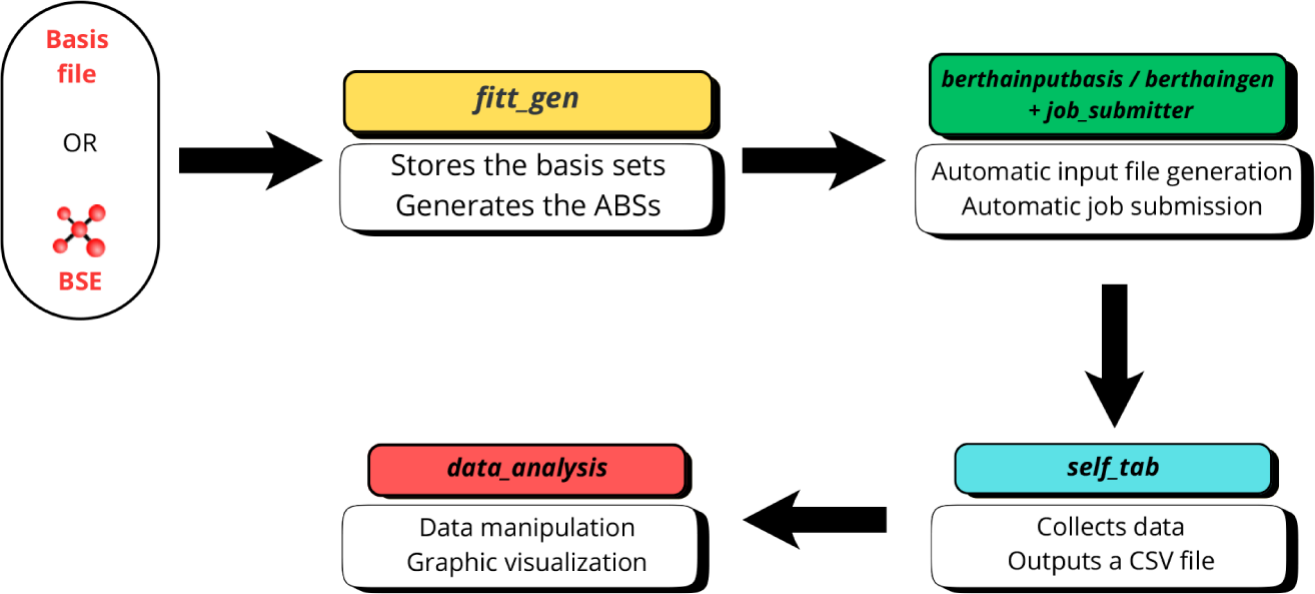
Workflow diagram of the implemented procedure to (i) generate ABS, (ii) construct BERTHA inputs and run them, (iii) collect data in CSV file and (iv) data analysis.

### 4.1 Molecular Data Set

Here, we briefly describe the molecular data set used to verify the accuracy of our automatically generated ABS. As mentioned earlier, due to the all-electron nature of our DKS calculations, our goal was to develop a method accurate enough for the entire periodic table, including the heavy and superheavy elements. This requires the testing of our automatic scheme on a large molecular data set representative of all groups and periods of the periodic table. Pollak and Weigend^25^ published a large molecular data set that included systems containing almost all elements of the periodic table except for the seventh period elements. We used this molecular data set as a starting point, removing those molecules with a multi configurational character which fail to converge in our DKS calculations. All the removed molecules contain *f* elements. For this reason, the molecules with *f* elements in the data set are limited to a few closed-shell molecules, namely: *CeH*_2_, *DyF*_2_, *La*_2_ *O*_3_, *LaF*_3_, *LaI*_3_ and *UF*_6_. The molecular data set has been further expanded to include elements from the seventh period and noble gases that were both missing in the original data set. We obtained the seventh-period molecules by replacing the heavy element in the sixth-period analogues with the corresponding element from the seventh period. The geometries for these systems were optimized using the ADF software^49^ at ZORA level including the spin–orbit coupling in combination with the BLYP exchange correlation functional and TZ2P basis set. In order to include noble gases, we use the complexes *OBeX* (X = *He*, *Ne*, *Ar*, *Kr*, *Xe*, *Rn*, *Og*). In these systems the *OBeX* molecule, with its very large dipole moment^50^ allows to strongly polarize the noble gases and thus is a perfect test for the ABS in these systems. The final data set included 286 molecules and covered the entire periodic table, with the exception of the *f* block, which, as already mentioned, are only sparsely represented due to their multireference character. The final version of the molecular data set includes all molecular geometries in XYZ format and is freely available at the public repository.^51^

## 5 Results and Discussion

The density-fitting scheme implemented in the DKS module of BERTHA ensures that the Coulomb energy in eq 17 is approximated from above and that the total energy is strictly approached from below. The latter is a consequence of the direct use of the fitted density in the evaluation of the exchange-correlation terms (see also ref. 37) We have already shown many years ago that for molecules containing heavy elements, very accurate results can be obtained with error in the Coulomb energy below 10^–4^-10^–5^ hartree (of the order of μhartree *per electron*). This error is of the same order of magnitude as in the nonrelativistic Kohn–Sham implementations^14,15,52,53^ that use density fitting for molecules with light elements or using pseudopotentials. As already mentioned, in our case the fitted density is also used for the evaluation of the exchange correlation term. This leads to errors in the total energy that are typically one or 2 orders of magnitude larger. Nonetheless, we have shown^5^ (see also Köster et al. in the nonrelativistic context^37^) that many chemical quantities (i.e., binding energies, harmonic frequencies, geometries and other properties) can be obtained in excellent agreement with exact calculations (i.e., without using the density fitting approximation) when simple correction schemes are applied (see ref. 5). In the Supporting Information we report an explicit example for the Au_2_ molecule. For our purposes here, we evaluate the accuracy of our ABS using the error in the Coulomb energy, defined as the difference between the Coulomb energies obtained using the exact density (*ρ*(r)) and that using the approximated fitted density (*ρ̃*(r)) , see eq 17.

We present the results obtained using Dyall’s basis sets, namely *dyall.v2z* and *dyall.v3z*.^54^ These basis sets are explicitly designed for all electron-relativistic atomic and molecular structure calculations and are available for all elements in the periodic table, including superheavy atoms. In conjunction with these principal basis sets, we tested the performance of four different auxiliary fitting basis sets. The latter were automatically generated with two different values for *n* and *l*_fitt_, namely GEN-n2-v1 (*n* = 2, *l*_fitt_ = *l*_temp_+1), GEN-n2-v2 (*n* = 2, *l*_fitt_ = *l*_temp_+2), GEN-n3-v1 (*n* = 3, *l*_fitt_ = *l*_temp_+1) and GEN-n3-v2 (*n* = 3, *l*_fitt_ = *l*_temp_+2). Each auxiliary fitting basis set was tested with the molecular data set described in the previous section (286 molecules). The results are shown in Figures 2–5. For each ABS, we give the absolute error in the total Coulomb energies (Δ*E*_*J*_, panel a) and the absolute error *per electron* (Δ*E*_*J*_/*electron*, panel b). The mean value and the corresponding standard deviation are also shown in the Figures (for the numerical values, see Tables S2 and S3).

**Figure 2:**
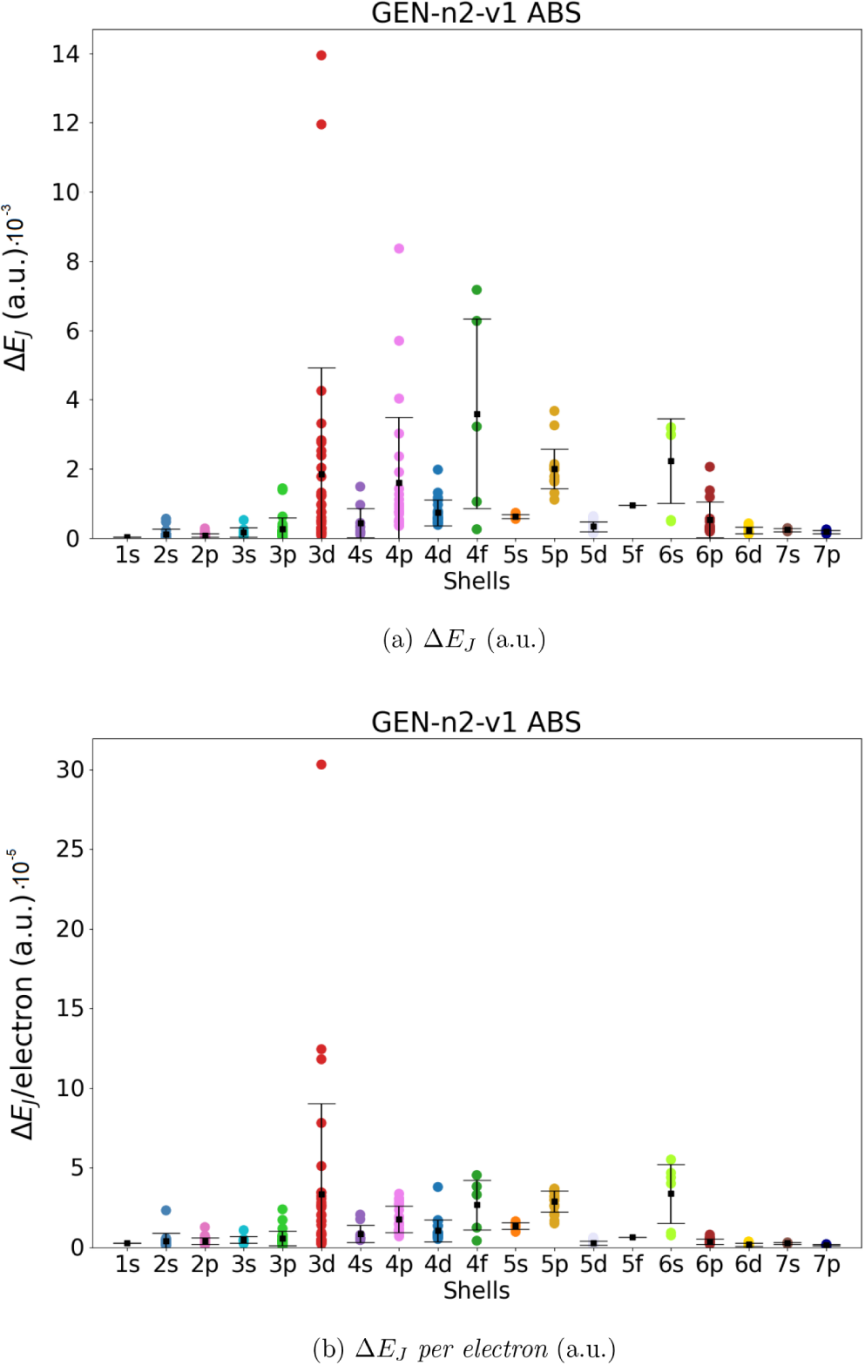
Coulomb energy error (Δ*E*_*J*_, panel a) and Coulomb energy error per electron (Δ*E*_*J*_/ *per electron*, panel b) for all molecules in the data set. Mean error (black squares) and standard deviation have been also reported. Data obtained using Dyall.v2z basis set in combination with the automated generated GEN-n2-v1 ABS.

**3:**
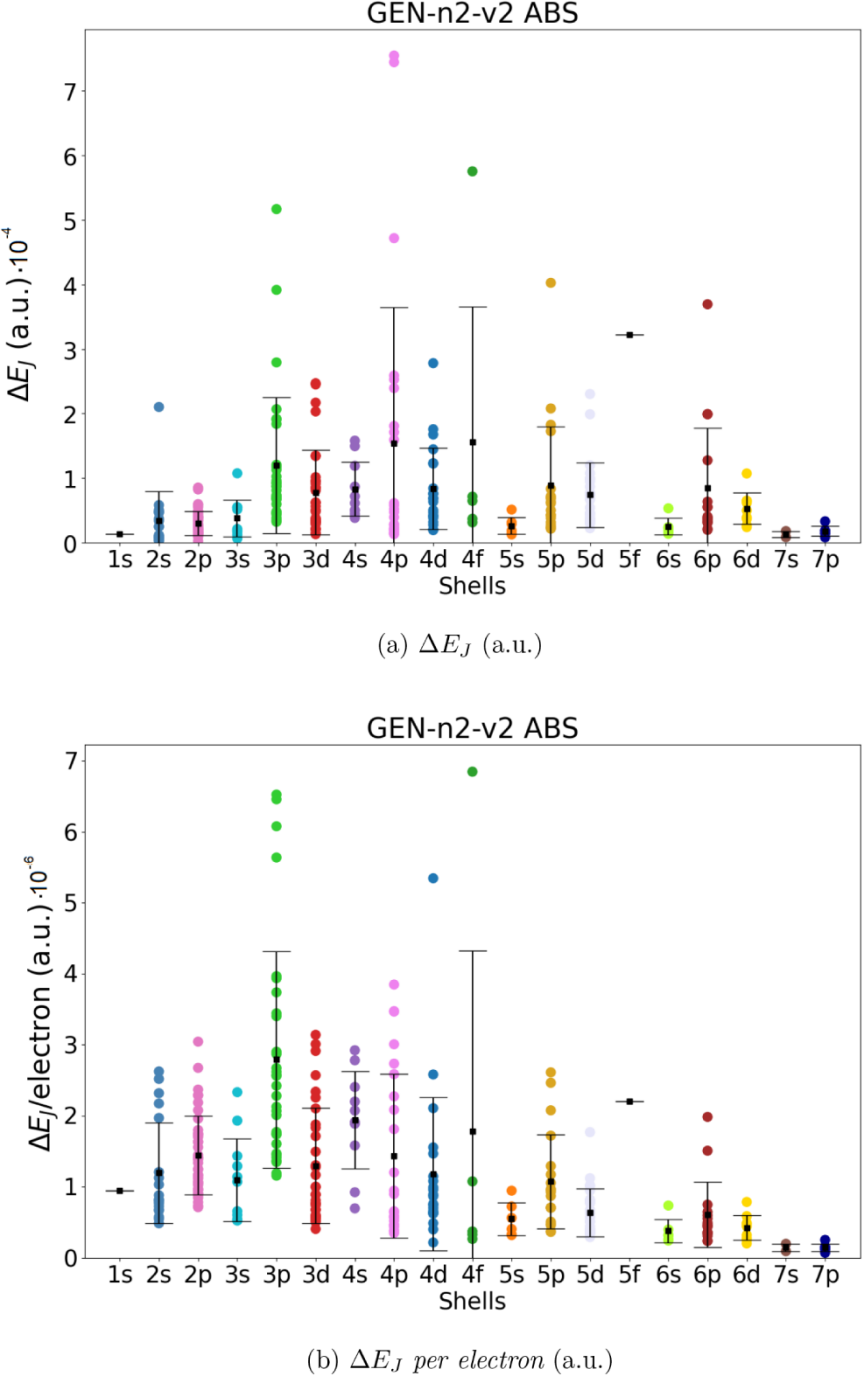
Coulomb energy error (Δ*E*_*J*_, panel a) and Coulomb energy error per electron (Δ*E*_*J*_/ *per electron*, panel b) for all molecules in the data set. Mean error (black squares) and standard deviation have been also reported. Data obtained using Dyall.v2z basis set in combination with the automated generated GEN-n2-v2 ABS.

**4:**
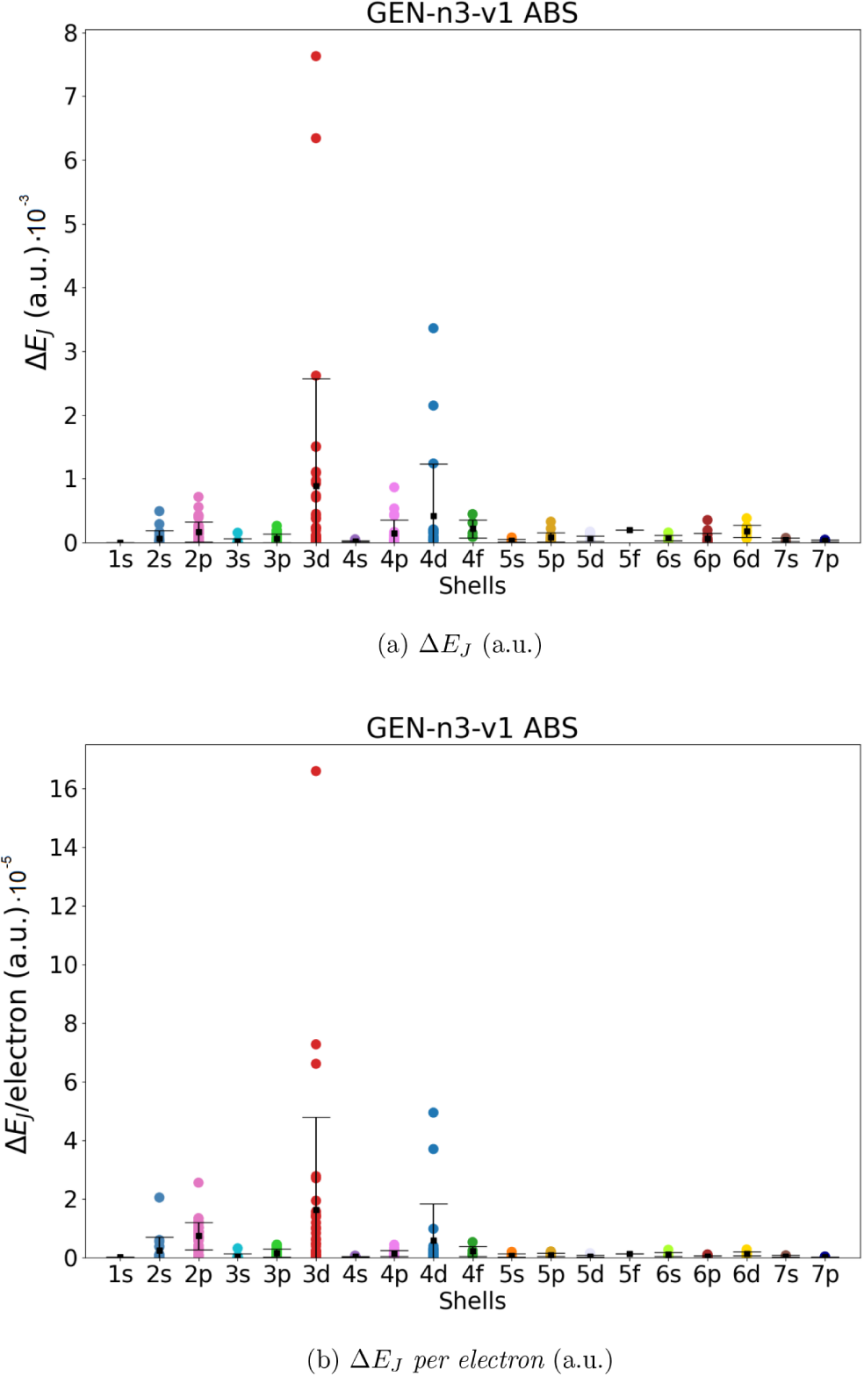
Coulomb energy error (Δ*E*_*J*_, panel a) and Coulomb energy error per electron (Δ*E*_*J*_/*per electron*, panel b) for all molecules in the data set. Mean error (black squares) and standard deviation have been also reported. Data obtained using Dyall.v2z basis set in combination with the automated generated GEN-n3-v1 ABS.

**Figure 5:**
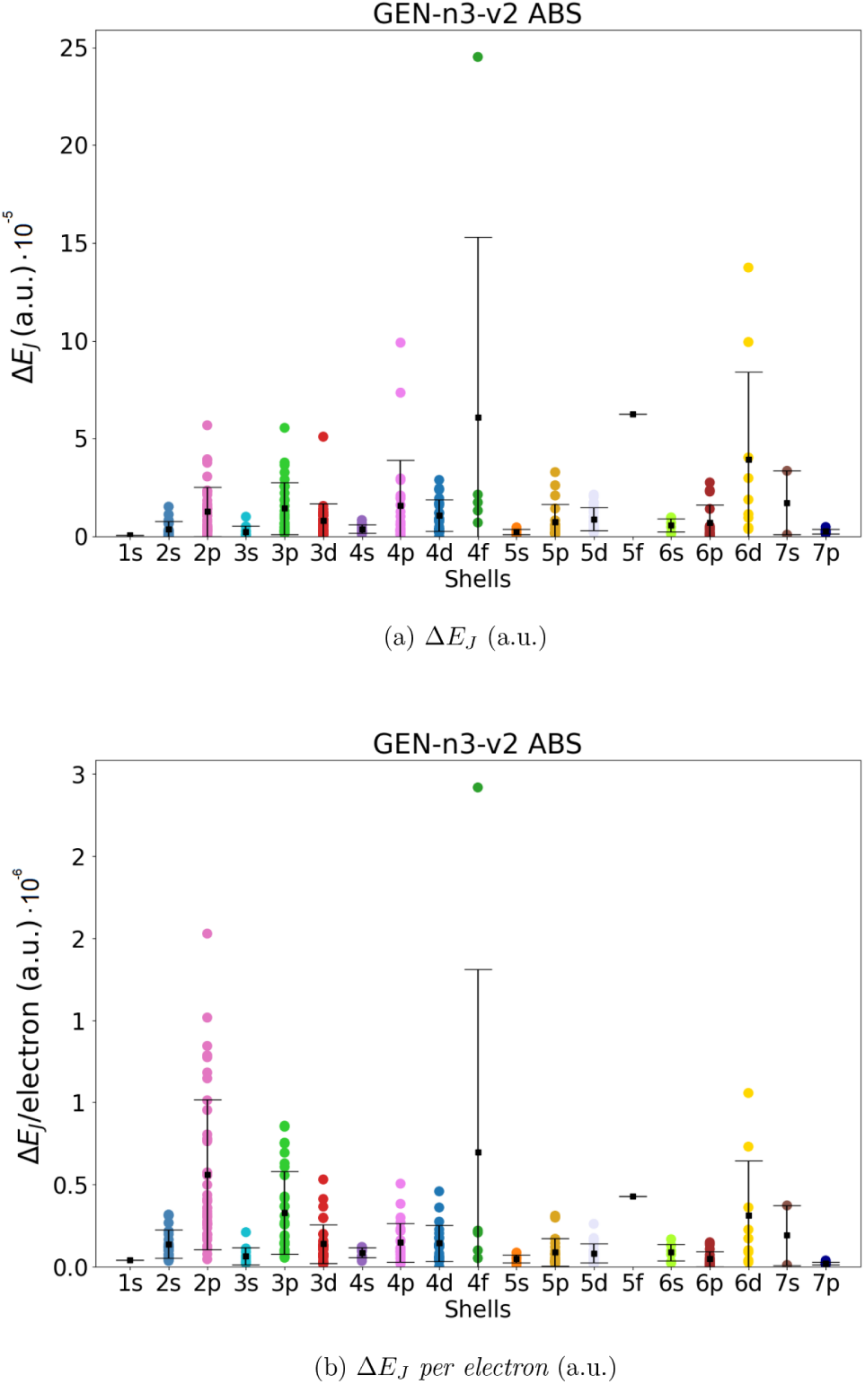
Coulomb energy error (Δ*E*_*J*_, panel a) and Coulomb energy error per electron (Δ*E*_*J*_/*per electron*, panel b) for all molecules in the data set. Mean error (black squares) and standard deviation have been also reported. Data obtained using Dyall.v2z basis set in combination with the automated generated GEN-n3-v2 ABS.

Our data show that the automatically generated ABS with the smaller value of *l*_fitt_ (lower angular flexibility), namely GEN-n2-v1 and GEN-n3-v1, are not entirely satisfactory. Although the mean value of Δ*E*_*J*_ is smaller than 3·10^–3^ and 9·10^–4^ hartree for all molecular groups (divided into shells), there are some molecular systems that exhibit errors up to 1.4·10^–2^ and 8·10^–4^ hartree, respectively, for GEN-n2-v2 and GEN-n3-v2. The accuracy increases significantly when ABS with higher angular flexibility are used (i.e., GEN-n2-v2 and GEN-n3-v2). In the case of GEN-n2-v2 we obtained a mean value of Δ*E*_*J*_ and Δ*E*_*J*_/*electron* always smaller than 8·10^–4^ hartree for all shells (in the worst case the error is of 1.6.10^–4^ hartree for the 4*f* shell). Using the largest GEN-n3-v2 basis set, we obtain an even higher accuracy, with an average error in Coulomb energy always below 6·10^–5^ (obtained for the 5f shell) and an absolute error below 2.5·10^–4^. In terms of error per electron, we can achieve an accuracy of 9·10^–8^ or 3·10^–6^ hartree.

For the sake of completeness, the results are further summarized in Figures 6 and 7 where we present the comparison of different auxiliary fitting basis sets visualized for each group of the molecular data set in the form of the mean value of Δ*E*_*J*_ and the mean value of Δ*E*_*J*_/*per electron*, respectively. The data are shown on a logarithmic scale.

**Figure 6:**
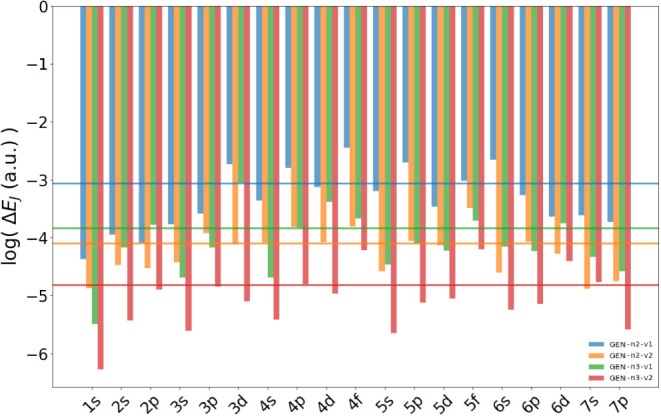
Mean value of Δ*E*_*J*_ in a.u. for each group of the molecular database. The data are obtained using different ABSs, namely GEN-n2-v1 (blue), GEN-n2-v2 (yellow), GEN-n3-v1 (green) and GEN-n3-v2 (red). The horizontal lines with the same colors represent the mean value of Δ*E*_*J*_ for all molecular systems. The data are shown in logarithmic scale.

**Figure 7:**
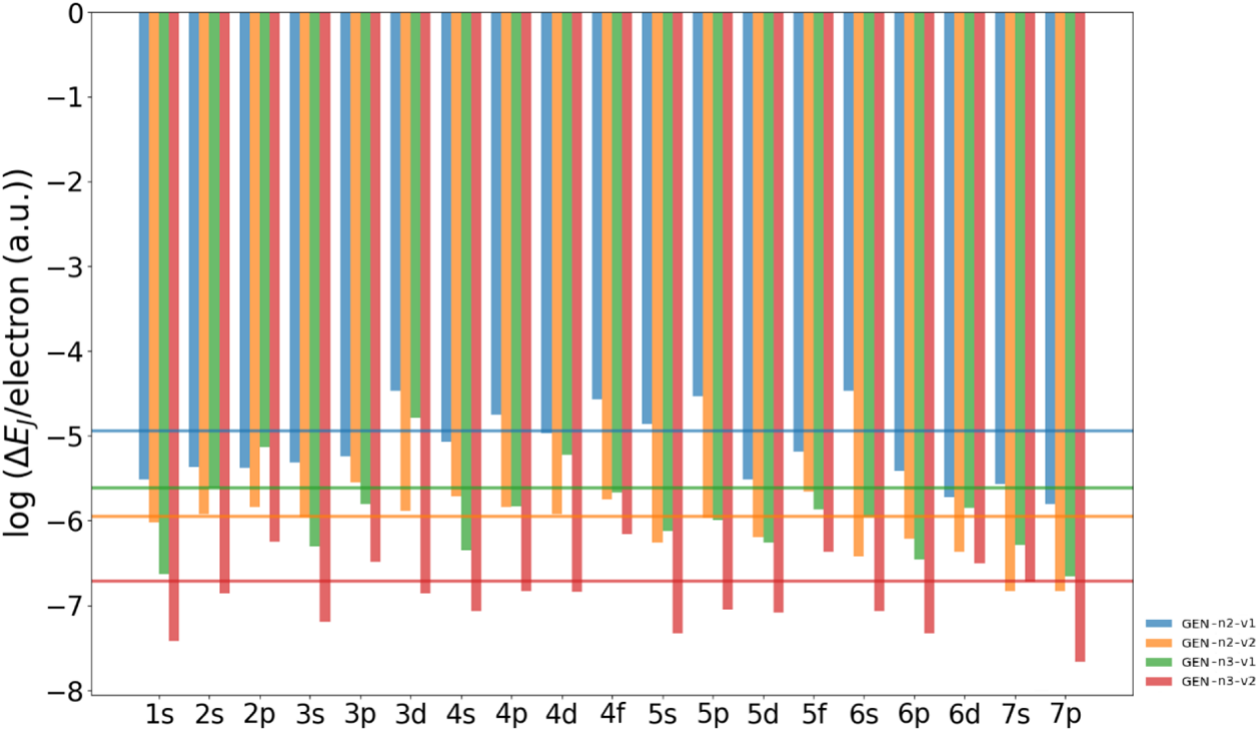
Mean value of Δ*E*_*J*_/*per electron* in a.u. for each group of the molecular data set. The data are obtained using different ABSs, namely GEN-n2-v1 (blue), GEN-n2-v2 (yellow), GEN-n3-v1 (green) and GEN-n3-v2 (red). The horizontal lines with the same colors represent the mean value of Δ*E*_J_/*per electron* for all molecular systems. The data are shown in logarithmic scale.

As mentioned above, one of the potential advantages of the automatic ABS generation process is that it can adapt to the main basis set used for the generation. In Figure 8, we clearly show that despite its simplicity, our automatic generation algorithm is able to provide accurate ABS even when we use a higher angular momentum principal basis set. The data show the comparison of the mean error of Δ*E*_*J*_ obtained with *dyall.v2z* and *dyall.v3z* in combination with the respective automatically generated GEN-n3-v1 basis sets. It is interesting to note that when switching from *dyall.v2z* to *dyall.v3z*, the accuracy is even slightly improved due to the higher angular flexibility of the generated GEN-n3-v1 basis set.

**Figure 8:**
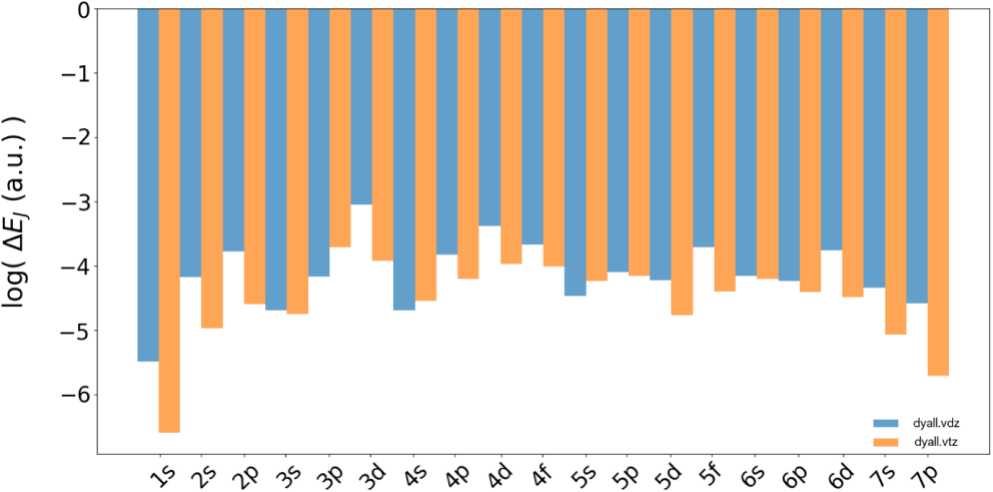
Comparison between the logarithms of the mean errors on Δ*E*_*J*_i n a.u. for the GEN-n3-v1 fitting set generated from the dyall.v2z (in blue) and dyall.v3z (in orange) basis sets.

To further asses our results it is interesting to compare the automatically generated fitting sets (namely GEN-n2-v1, GEN-n2-v2, GEN-n3-v1 and GEN-n3-v2) with respect to the two auxiliary basis sets that we optimized for gold atom^4^ named *B*16 and *B*20. Indeed, in Table 1 we show the results of the density fitting calculations using the four different ABSs generated from the *dyall.v2z* and the ones obtained using the two cited *B*16 and *B*20 sets. As a reference, the results obtained with the conventional (without density fitting) DKS calculation of the Au_2_ molecule are reported (this is referred as ″Exact″ in the table). For completeness, in the table we also report the experimental values.^55,56^ For the equilibrium bond length of Au_2_, the results show that the fitting method essentially agrees with the calculations carried out without using the density fitting scheme, regardless of the auxiliary basis used. The largest discrepancy exists for the smallest basis (GEN-n2-v1), which underestimates the bond length by 0.004 Å. The agreement for other spectroscopic parameters is also excellent: the error for the harmonic frequency is 1 cm^–1^ with the GEN-n2-v1 and almost exact values for the bases GEN-n2-v2, GEN-n3-v2 and B20. Similarly, the effect of using the density fitting is almost negligible (error of 0.001 eV) when the GEN-n3-v2 and B20 basis sets are used.

**Table 1:** Spectroscopic Constants in the Au Dimer Calculated at the DKS/BLYP Using the dyall.V2z Basis Set in Combination with Several Auxiliary Density Fitting Basis Set^a^

	B16	B20	GEN-n2-v1	GEN-n2-v2	GEN-n3-v1	GEN-n3-v2	Exact	Exp.
R_*e*_ (Å)	2.543	2.543	2.539	2.543	2.544	2.543	2.543	2.472
ω_*e*_ (cm^–1^)	171.0	169.6	170.5	169.6	169.1	169.4	169.5	191
D_*e*_ (eV)	2.330^b^	2.325^b^	2.389^b^	2.314^b^	2.335^b^	2.323^b^	2.324^b^	2.36
Δ*E*_*J*_ (a.u.)	0.000094	0.000006	0.000345	0.000055	0.000026	0.000002		

a The Absolute Error (ΔEJ) in the Coulomb and the Values Obtained Using the Implementation without the Density Fitting Algorithm (exact) are Also Reported. The Experimental Data (exp.) Have Been Obtained from refs. 55,56 .

b The covalent dissociation energy was determined by calculating the atomic closed-shell monoions (cation and anion) and employing the experimental values of the ionization potential and electron affinity of gold.

An obvious disadvantage of automatically generated ABS is that they contain a larger number of functions than those found for specially optimized sets. This affects the computational efficiency, which is the main goal when the density fitting scheme is introduced. In Table 2 we show that, as expected, the automatically generated ABS are generally larger than the optimized ones.

**Table 2:** Size of Auxiliary Fitting Basis Set, CPU Times (S), Speed Ratio and Accuracy in Coulomb Energy (Mhartree) for Gold Dimer (Au_2_)­^a^

ABS	Size	Time (s)	Speed-up	Δ*E*_*J*_
B16	420	11	128	0.094
B20	614	14.8	95	0.006
GEN-n2-v1	554	12.5	113	0.345
GEN-n2-v2	944	19.4	73	0.055
GEN-n3-v1	734	16.3	86	0.026
GEN-n3-v2	1248	25.8	54	0.002

a Speed-Up is Calculated with Respect the Calculation without Density Fitting Algorithm. DKS/BLYP Using the Dyall.V2z Basis Set. The Absolute Error in the Coulomb and in the Total Energy are Also Reported. All Runs Done with Intel Xeon CPU E5–2683 V4 2.1.

The largest ABS, GEN-n3-v2, achieves an error that is one-third of the error achieved with B20, but at the cost of approximately doubling the number of fitting functions and consequently increasing the computational effort. With B20, the increase in speed (i.e., Speed-up) compared to the calculation without density fitting is slightly less than 100, while with GEN-n3-v2 it is slightly more than 50. If, on the other hand, the similarly sized B16 and GEN-n2-v1 ABS are compared, it becomes clear that the error in the Coulomb energy of GEN-n2-v1 is about an order of magnitude higher, although the acceleration is almost identical. The GEN-n3-v2 basis set offers the highest accuracy but it is reasonable to argue that for those seeking a balance between cost and accuracy, GEN-n3-v1 is probably a good compromise, at least for this system. Although our ABSs have not yet reached the computational efficiency of B20 (which benefits from less, well-optimized fitting functions), it is noteworthy that the generation of ABSs for the entire periodic table for a given atomic principal spinor takes less than one second.

The numerical stability of the solution of the linear systems containing the matrix A (the Coulomb matrix of the two-electron integrals of the auxiliary functions, see eqs 18 and 23) is a subtle point to consider. Although the matrix A is in principle positive definite, it may be ill-conditioned due to finite precision arithmetic. The condition number indicates how sensitive the solution of a linear system is to small changes in the input data or the matrix itself. A matrix with a lower condition number is better conditioned, i.e., the numerical solutions are probably more stable. Conversely, a high condition number indicates a poorly conditioned matrix where the solutions are very sensitive to perturbations, which can lead to numerical instability in the density fitting scheme. As mentioned in the literature, the automatically generated ABS can be particularly susceptible to this problem and therefore a numerical scheme can be introduced to avoid this problem (see Köster et al.^17^ and references therein for a detailed description). In the case of the Au_2_ molecule, we evaluate the condition numbers of the matrix A for all different ABSs. Their values are very high in all cases: about 10^20^ and 10^22^ for *B*16 and *B*20 and, as expected, even higher, 10^25^, 10^28^, 10^26^ and 10^30^ for the automatically generated GEN-n2-v1, GEN-n2-v2, GEN-n3-v1 and GEN-n3-v2 respectively. The higher values of the condition numbers are for the automatically generated ABS. Interestingly, despite the high condition numbers, we did not observe any numerical noise during the SCF procedure for the entire set of 286 molecules. Moreover, the condition numbers associated with systems that were excluded from the data set due to the intrinsic limitations of DFT in describing multiconfigurational electronic structures, do not significantly deviate from the general behavior observed across the full data set. We do not observe abnormally large condition numbers in these excluded cases. For instance, osmium-containing molecules (included in the final data set) exhibit condition numbers on the order of 10^27^, while praseodymium-containing molecules (excluded during data set pruning) display values around 10^28^. As a more rigorous proof of the numerical stability, we tested our automatically generated ABSs in a real-time TDDKS simulation. We use the PyBERTHART code,^27^ the Python API for the integral kernel of BERTHA. The real-time propagation of the DKS equation is extremely sensitive to any source of numerical problems. Indeed, at each step of the time evolution, the DKS matrix must be evaluated many times and the total number of time steps can be in the order of thousands.

In Figure 9 we show the absorption spectrum of the PbCl_2_ molecule using the dyall.2vz basis in combination with different automatically generated ABSs. The spectrum was obtained by Fourier transforming the time-dependent dipole moment induced by a kick perturbation using a Gaussian damping function with an exponent of 1.10^–6^. The simulation was performed with a time step of 0.1 au for a total simulation time of 72.5 fs (i.e., 30000 time steps). Regardless of the ABS used, we observed very stable propagation both in terms of induced dipole and total energy conservation. The resulting absorption spectrum is very clean, without the numerical artifacts that typically occur with numerical instability of the time-evolution procedure.

**Figure 9:**
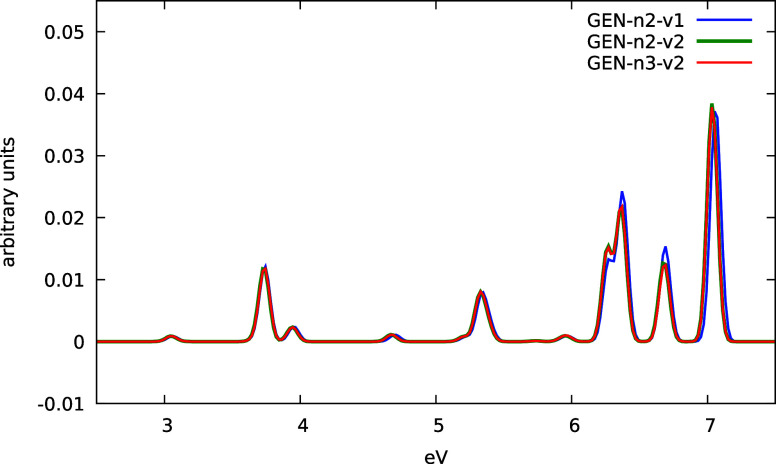
Absorption spectra of the PbCl_2_ molecule using different automatic generated ABS. See text for details.

## 6 Conclusions

The development of efficient algorithms based on density fitting is an active area of research as it can be crucial to reduce the computational cost of electronic structure methods in quantum chemistry. While this approach is well established in the nonrelativistic regime, its full applicability in a four-component relativistic DKS framework remains a challenge, in particular due to the lack of well-optimized auxiliary basis sets (ABSs) for the heaviest elements of the periodic table.

In this work, we have developed a fully automatic and modular workflow for the generation of ABS in the framework of a relativistic 4-component code, starting from some information (exponents and angular distribution) obtained from the basis sets of the main atomic spinors. We have generated auxiliary basis sets of different sizes and accuracies and tested their performance in the DKS module of the BERTHA code using a large molecular data set (almost 300 molecules with elements from H to Og). With the largest auxiliary basis set, we can achieve an accuracy in the Coulomb energy even of less than a μHartree. This is comparable to the accepted errors for the nonrelativistic case. The choice of principal basis set has a significant impact on the size of the ABS, which consequently affects the computational cost and efficiency. Remarkably, our approach maintains high accuracy over the entire periodic table with minimal deviations and remains stable even when large principal basis sets are used (e.g., the basis set dyall.vtz).

A key advantage of our workflow is its automation, which enables the rapid creation, testing and refinement of auxiliary basis sets. This makes it a valuable tool for future developments in the field of new density auxiliary basis sets generation. While our results show high accuracy, our preliminary analysis shows that automatically generated ABS generally have higher condition numbers than fitting basis sets that have been specifically optimized according to energetic criteria. Our future work should focus on improving the algorithm for the automatic generation of exponents. This could potentially lead to a further reduction in the size of such auxiliary basis sets and help avoiding potential numerical instabilities by using the reduction of the condition number as an additional criterion. The algorithm proposed here is accurate for the DKS calculations with local (LDA) and semilocal (GGA) exchange correlation functionals. However, it is known that high accuracy for Fock exchange requires auxiliary basis sets with much higher angular flexibility.^12,17^ Recently, Köster et al.^17^ developed an automatic generation of auxiliary basis sets of Hermite-Gaussian Type functions that are accurate for Hartree–Fock and DFT using hybrid functions for all elements up to Kr. The automatic workflow presented here forms the basis for future research into optimal auxiliary basis sets to extend four-component DKS to efficiently incorporate exact Fock exchange.

## Supplementary Material



## Notes

Molecules, grouped in shells, used to validate the automated generated auxiliary basis sets (PDF)

## References

[1] Sunaga A., Salman M., Saue T. (2022). 4-component relativistic Hamiltonian
with effective QED potentials for molecular calculations. J. Chem. Phys..

[2] Nonn A., Margócsy A., Mátyus E. (2024). Bound-State Relativistic Quantum
Electrodynamics: A Perspective for Precision Physics with Atoms and
Molecules. J. Chem. Theory Comput..

[3] Bloom B. P., Paltiel Y., Naaman R., Waldeck D. H. (2024). Chiral Induced Spin
Selectivity. Chem. Rev..

[4] Belpassi L., Tarantelli F., Sgamellotti A., Quiney H. M. (2006). Electron density
fitting for the Coulomb problem in relativistic density-functional
theory. J. Chem. Phys..

[5] Belpassi L., Tarantelli F., Sgamellotti A., Quiney H. M. (2008). All-electron four-component
Dirac-Kohn-Sham procedure for large molecules and clusters containing
heavy elements. Phys. Rev. B.

[6] Storchi L., Belpassi L., Tarantelli F., Sgamellotti A., Quiney H. M. (2010). An Efficient Parallel All-Electron
Four-Component Dirac-
Kohn- Sham Program Using a Distributed Matrix Approach. J. Chem. Theory Comput..

[7] Storchi L., Rampino S., Belpassi L., Tarantelli F., Quiney H. M. (2013). Efficient parallel all-electron four-component Dirac–Kohn–Sham
program using a distributed matrix approach II. J. Chem. Theory Comput..

[8] Rampino S., Belpassi L., Tarantelli F., Storchi L. (2014). Full parallel implementation
of an all-electron four-component dirac–kohn–sham program. J. Chem. Theory Comput..

[9] Belpassi L., De Santis M., Quiney H. M., Tarantelli F., Storchi L. (2020). BERTHA: Implementation of a four-component Dirac-Kohn-Sham
relativistic framework. J. Chem. Phys..

[10] Storchi L., Bellentani L., Hammond J., Orlandini S., Pacifici L., Antonini N., Belpassi L. (2025). Acceleration of the
Relativistic Dirac-Kohn-Sham Method with GPU: A Pre-Exascale Implementation
of BERTHA and PyBERTHA. J. Chem. Theory Comput..

[11] Konecny L., Kadek M., Komorovsky S., Ruud K., Repisky M. (2018). Resolution-of-identity
accelerated relativistic two- and four-component electron dynamics
approach to chiroptical spectroscopies. J. Chem.
Phys..

[12] Kelley M. S., Shiozaki T. (2013). Large-scale Dirac-Fock-Breit
method using density fitting
and 2-spinor basis functions. J. Chem. Phys..

[13] Quiney H. M., Belanzoni P., Sgamellotti A. (2002). Evaluation of the Coulomb energy
in relativistic self-consistent-field theory. Theor. Chem. Acc..

[14] Lehtola S. (2021). Straightforward
and Accurate Automatic Auxiliary Basis Set Generation for Molecular
Calculations with Atomic Orbital Basis Sets. J. Chem. Theory Comput..

[15] Lehtola S. (2023). Automatic
Generation of Accurate and Cost-Efficient Auxiliary Basis Sets. J. Chem. Theory Comput..

[16] Stoychev G. L., Auer A., Neese F. (2017). Automatic Generation
of Auxiliary
Basis Sets. J. Chem. Theory Comput..

[17] Dïaz-Tinoco M., Flores-Moreno R., Zúñiga-Gutiérrez B. A., Köster A. M. (2025). Automatic
Generation of Even-Tempered Auxiliary Basis
Sets with Shared Exponents for Density Fitting. J. Chem. Theory Comput..

[18] Aquilante F., Gagliardi L., Pedersen T. B., Lindh R. (2009). Atomic Cholesky
decompositions:
a route to unbiased auxiliary basis sets for density fitting approximation
with tunable accuracy and efficiency. J. Chem.
Phys..

[19] Aquilante F., Lindh R., Pedersen T. B. (2007). Unbiased
auxiliary basis sets for
accurate two-electron integral approximations. J. Chem. Phys..

[20] Aquilante F., Pedersen T. B., Lindh R., Roos B. O., de Merás A. S., Koch H. (2008). Accurate ab initio
density fitting for multiconfigurational self-consistent
field methods. J. Chem. Phys..

[21] Hellmann L., TÖlle J., Niemeyer N., Neugebauer J. (2022). Automated
Generation of Optimized Auxiliary Basis Sets for Long-Range-Corrected
TDDFT Using the Cholesky Decomposition. J. Chem.
Theory Comput..

[22] Yang R., Rendell A. P., Frisch M. J. (2007). Automatically generated
Coulomb fitting
basis sets: Design and accuracy for systems containing H to Kr. J. Chem. Phys..

[23] Zhang C., Lipparini F., Stopkowicz S., Gauss J., Cheng L. (2024). Cholesky Decomposition-Based
Implementation of Relativistic Two-Component Coupled-Cluster Methods
for Medium-Sized Molecules. J. Chem. Theory
Comput..

[24] Banerjee S., Zhang T., Dyall K. G., Li X. (2023). Relativistic resolution-of-the-identity
with Cholesky integral decomposition. J. Chem.
Phys..

[25] Pollak P., Weigend F. (2017). Segmented Contracted
Error-Consistent Basis Sets of
Double- and Triple-*ζ* Valence Quality for One-
and Two-Component Relativistic All-Electron Calculations. J. Chem. Theory Comput..

[26] Storchi, L. ; De Santis, M. ; Belpassi, L. REL 1.0.0 within the PyBertha project: https://github.com/BERTHA-4c-DKS/pybertha.

[27] De
Santis M., Storchi L., Belpassi L., Quiney H. M., Tarantelli F. (2020). PyBERTHART: A relativistic real-time four-component
TDDFT implementation using prototyping techniques based on Python. J. Chem. Theory Comput..

[28] Quiney H. M., Belanzoni P. (2002). Relativistic density functional theory
using Gaussian
basis sets. J. Chem. Phys..

[29] Belpassi L., Storchi L., Quiney H. M., Tarantelli F. (2011). Recent Advances
and Perspectives in Four-Component Dirac-Kohn-Sham Calculations. Phys. Chem. Chem. Phys..

[30] Grant I. P., Quiney H. M. (2000). Rayleigh-Ritz Approximation of the Dirac Operator in
Atomic and Molecular Physics. Phys. Rev. A.

[31] Relativistic quantum theory of atoms and molecules: Theory and computation. Springer series on atomic, optical, and plasma physics, Grant, I. P. Ed.; Springer: Berlin, 2007.

[32] Faegri, K. ; Dyall, K. G. Relativistic Electronic Structure Theory. Elsevier, 2002, pp. 259–290.

[33] Quiney H., Skaane H., Grant I. (1997). Relativistic
calculation of electromagnetic
interactions in molecules. J. Phys. B: At. Mol.
Opt..

[34] McMurchie L. E., Davidson E. R. (1978). One- and two-electron
integrals over cartesian gaussian
functions. J. Comput. Phys..

[35] Dunlap B. I., Connolly J. W. D., Sabin J. R. (1979). On some
approximations in applications
of X*α* theory. J. Chem.
Phys..

[36] Laikov D.
N. (1997). Fast evaluation
of density functional exchange-correlation terms using the expansion
of the electron density in auxiliary basis sets. Chem. Phys. Lett..

[37] Köster A. M., Reveles J. U., Del Campo J. M. (2004). Calculation
of exchange-correlation
potentials with auxiliary function densities. J. Chem. Phys..

[38] Birkenheuer U., Gordienko A., Nasluzov V., Fuchs-Rohr M., Rösch N. (2005). Model density approach to the Kohn-Sham problem: Efficient
extension of the density fitting technique. Int. J. Quantum Chem..

[39] Dunlap B. I., Palenik M. C. (2016). Variationally fitting the total electron-electron interaction. Phys. Rev. B.

[40] Köster A. (1996). Efficient
recursive computation of molecular integrals for density functional
methods. J. Chem. Phys.

[41] Köster A. (2003). Hermite Gaussian
auxiliary functions for the variational fitting of the Coulomb potential
in density functional methods. J. Chem. Phys..

[42] Alvarez-Ibarra A., Köster A., Zhang R., Salahub D. (2012). Asymptotic Expansion
for Electrostatic Embedding Integrals in QM/MM Calculations. J. Chem. Theory Comput..

[43] Koster, A. M. ; Geudtner, G. ; Alvarez-Ibarra, A. ; Calaminici, P. ; Casida, M. E. ; Carmona-Espindola, J. ; Dominguez, V. D. ; Flores-Moreno, R. ; Gamboa, G. U. ; Goursot, A. , deMon2k, Version 5; The deMon developers, 2018.

[44] Calaminici P., Janetzko F., Köster A. M., Mejia-Olvera R., Zuniga-Gutierrez B. (2007). Density functional
theory optimized basis sets for
gradient corrected functionals: 3d transition metal systems. J. Chem. Phys..

[45] Antonini, N. ; Belpassi, L. ; Storchi, L. Auxiliary basis set generator for the BERTHA code. https://github.com/BERTHA-4c-DKS/fitting_basis_generator. 10.5281/zenodo.15829790

[46] Pritchard B. P., Altarawy D., Didier B., Gibson T. D., Windus T. L. (2019). New basis
set exchange: An open, up-to-date resource for the molecular sciences
community. J. Chem. Inf. Model..

[47] Storchi, L. Input basis sets utility for BERTHA code. https://github.com/BERTHA-4c-DKS/berthainputbasis.

[48] Storchi, L. Input file generator module for the BERTHA code. https://github.com/BERTHA-4c-DKS/berthaingen.

[49] Te
Velde G., Bickelhaupt F. M., Baerends E. J., Fonseca
Guerra C., van Gisbergen S. J. A., Snijders J. G., Ziegler T. (2001). Chemistry
with ADF. J. Comput. Chem..

[50] Nunzi F., Cesario D., Pirani F., Belpassi L., Frenking G., Grandinetti F., Tarantelli F. (2017). Helium Accepts Back-Donation In Highly
Polar Complexes: New Insights into the Weak Chemical Bond. J. Phys. Chem. Lett..

[51] Molecular dataset. https://github.com/BERTHA-4c-DKS/bertha_testbench. 10.5281/zenodo.15829790

[52] Eichkorn K., Treutler O., Öhm H., Häser M., Ahlrichs R. (1995). Auxiliary basis sets to approximate Coulomb potentials. Chem. Phys. Lett..

[53] Manby F. R., Knowles P. J., Lloyd A. W. (2001). The Poisson equation
in density fitting
for the Kohn-Sham Coulomb problem. J. Chem.
Phys..

[54] Dyall, K. G. Dyall dz, tz, and qz basis sets for relativistic electronic structure calculations; Zenodo, 2023.

[55] Simard B., Hackett P. (1990). High resolution study of the (0,
0) and (1, 1) bands
of the A0u+-X0g+ system of Au2. J. Mol. Spectrosc..

[56] Huber, K. P. ; Herzberg, G. Molecular Structure IV, Constants of Diatomic Molecules; Van Nostrand Reinhold: New York, 1979.

