# Supplementary material for: Automatic Generation of Density-Fitting Auxiliary Basis Sets for All-Electron Dirac–Kohn–Sham Calculations
Source: J Phys Chem A. 2025 Jul 16;129(30):6930–41. doi: 10.1021/acs.jpca.5c02772 (PMC12319901; doi:10.1021/acs.jpca.5c02772)
Supplement: Supplementary file 1 [file jp5c02772_si_001.pdf]

Supporting Information for Publication: Automatic generation of density fitting auxiliary basis sets for all electron Dirac-Kohn-Sham calculations.

Nicoló Antonini,[†,‡] Enrico Ronca,[‡] Loriano Storchi,[*,†,¶] and Leonardo Belpassi[*,¶]

†*Dipartimento di Farmacia, Università G. d'Annunzio Chieti-Pescara, via dei Vestini, 66100 Chieti, Italy*

‡*Dipartimento di Chimica, Università degli Studi di Perugia, via Elece di Sotto 8, 06123 Perugia, Italy*

¶ *CNR Institute of Chemical Science and Technologies "Giulio Natta" (CNR-SCITEC), via Elce di Sotto 8, 06123 Perugia, Italy*

E-mail: loriano@storchi.org; leonardo.belpassi@cnr.it

1 Molecular Dataset

In the following we report the molecules, grouped in shells, used to validate the automated generated auxiliary basis sets. Geometries (in XYZ format) are available in the GitHub repository (https://github.com/BERTHA-4C-DKS/bertha_testbench).

1s

$H_2, OBeHe$

2s

$Be_2F_4, Be_2H_4, Be_4, BeC_2H_6, BeF_2O_2H_4, BeH_2, BeS, Li_2O,$

$Li_2, Li_4C_4H_{12}, Li_4Cl_4, Li_4H_4, Li_8, LiBH_4, LiCl, LiF, LiH, LiSLi, PLi_3$

2p

$B_2H_6, B_3N_3H_6, B_4H_4, BF_3, BH_3CO, BH_3NH_3, BH_3, C_2H_2, C_2H_3N, C_2H_4, C_2H_6, C_4H_4,$

$C_6H_6, CF_4, CH_2O_2, CH_2O, CH_3N, CH_3OH, CH_4, CO_2, CO, F_2, H_2CO_3,$

$H_2O_2, H_2O, HCN, HF, HNC, HNO_2, HNO_3, HNO, N_2H_2, N_2H_4, N_2, N_4,$

$NF_3, NH_3, NH_4F, OBeNe, OF_2$

3s

$Mg_4, MgCl_2, MgF_2, MgH_2, Na_2O, Na_2S, Na_3N, Na_3P, NaCl, NaF, NaH$

3p

$Al_2O_3, Al_2S_3, AlCl_3, AlF_3, AlH_3, AlN, Cl_2, ClF_3, ClF, CS_2,$

$H_2SO_4, H_3PO_4, HCl, HCP, HSH, HSSH, OBeAr, P_2, P_4, PF_3, PF_5,$

$PH_3, S_5, SF_2, SF_4, SF_6, SiCl_4, SiF_4, SiH_4, SiO_2, SiS_2$

4s

$CaCl_2, CaF_2, CaH_2, K_2S, K_3P, KBr, KCl, KF, KH, KI$

3d

$CoCl_3, CoF_3, Cr(CO)_6, CrO_3, Cu_2O, Cu_2S, Cu_2, CuCl, CuF, CuH, Fe(CO)_5, FeF_2, FeO,$

$Ferrocen, Ni(CO)_4, NiF_2, NiO, ScCl_3, ScF_3, ScH_3, TiCl_4, Ti(CO)_4,$

$TiF_4, TiH_4, TiO_2, TiO, TiS_2, VOF_3, ZnCl_2, ZnF_2, ZnH_2, ZnMe_2$

4p

$As_4S_4, As_4, AsCl_3, AsCl_6^{-1}, AsH_3, Br_2, BrCl, BrO_4^{-1}, GaCl_3, GaCl,$
$GaF, GaH_3, GeCl_4, GeF_4, GeH_4, GeO_2, GeO, HBr, HCBr_3, OBeKr, Se_8, SeH_2, SeO_2, SeO$

5s

$RbH, SrO, SrH_2, SrS, RbF, SrF_2$

4d

$Ag_2, AgCl, CdF_2, CdMe_2, Mo(CO)_6, MoO_2, MoO_3, NbF_3, NbO_2F, Pd(CO)_4,$
$RhF, Ru(CO)_5, RuO_2, RuO_4, RuO, Tc_2O_7, TcO_3F, YF_3, YF, ZrO_2, ZrO$

5p

$I_2, ICl, IH, InCl_3, InCl, InH_3, InH, IO_4^{-1}, OBeXe, SbCl_6^{-1}, SbF_3, SbF,$
$SbH_3, SnH_4, SnO_2, SnO, TeH_2, TeO_2, TeO$

6s

$BaF_2, BaH_2, BaO, BaS, CsF, CsH$

4f

$CeH_2, DyF_2, La_2O_3, LaF_3, LaI_3$

5d

$Au_2, Au_3^{-1}, AuCl_3, AuCl, AuH, Hg_2Cl_2, HgF_2, HgMe_2, Os(CO)_5, OsO_2, OsO_3, OsO_4,$
$Pt(CO)_4, PtO_2, PtO, ReH, ReO_3F, TaF_3, TaF, TaH_5, TaO_2F, W(CO)_6, WO_2, WO_3, WO$

6p

$At_2, Bi_4, BiCl_6^{-1}, BiF_3, BiF, BiH_3, OBeRn, PbH_4, PbO_2, PbO, PoO_2, PoO, TlCl_3, TlCl, TlH_3, TlH$

7s

FrH, RaH_2

5f

UF_6

6d

$BhH, DsO, DbH_5, SgO, RgH, MtF_3, RfF_4, HsO_3, CnF_2$

7p

$FlH_4, LvO, McH_3, NhH, OBeOg, TsH$

2 Energy

The variational density fitting in BERTHA uses the Coulomb metric, which ensures that the Coulomb energy is approximated from below in the fitting procedure, and we can easily achieve an accuracy of $10^{-4}-10^{-5}$ hartree in terms of absolute energy in our DKS calculation for all electrons, which is of the same order of magnitude as that achievable with the non-relativistic Kohn-Sham implementation.

The Table S1 presents spectroscopic properties of the gold dimer, including equilibrium bond length (R_e), harmonic frequency (ω_e), and dissociation energy (D_e), using two auxiliary basis sets, `B16` and `B20`[1] , containing 210 and 307 Hermite-Gaussian functions per atom, respectively. These basis sets were optimized using the Coulomb variational scheme for gold.[2]

The principal G-spinor basis set for each gold atom was the all-electron Dyall.v2z basis, with the corresponding small component basis generated using the restricted kinetic balance relation. The exchange-correlation functional employed was the Becke 1988 (B88) exchange functional combined with the Lee-Yang-Parr (LYP) correlation functional (BLYP). All calculations were performed with a total energy convergence threshold of 10^{-7} hartree, and the

equilibrium bond length was determined iteratively through a quadratic fit to the energy.

Table S1 compares the results of the density-fitting calculations respect to those obtained using the conventional exact DKS Coulomb matrix. For the equilibrium bond length of Au_2, the results indicate that the fitting method essentially matches the exact result, irrespective of the auxiliary basis used. The agreement is also excellent for other parameters: the error in the harmonic frequency is 2 cm^{-1} with the `B16` basis and only 0.1 cm^{-1} with the `B20` basis. Similarly, the dissociation energy is nearly exact when the `B20` basis is employed.

Table S1: Spectroscopic constants for the Au dimer calculated at the DKS/BLYP level using the Dyall.v2z basis set with various auxiliary density-fitting basis sets. Absolute errors in the Coulomb and total energy are also provided. Values in parentheses correspond to calculations corrected by adding the term $(E^{(l)}_{XC}[\rho(\mathbf{r})] - E^{(l)}_{XC}[\tilde{\rho}(\mathbf{r})])$ to Eq. S2.

	B16	*B20*	Exact
R_e (Å)	2.543	2.543	2.543
	(2.542)	(2.543)	
ω_e (cm^{-1})	171.0	169.6	169.5
	(169.7)	(169.4)	
D_e (eV)	2.330	2.325	2.324
	(2.324)	(2.324)	
ΔE_J (hartree)	0.000094	0.000006	
ΔE_{tot} (hartree)	0.030440	0.004875	
	(0.000009)	(0.000008)	

Nevertheless, looking at the results it is important to note that: despite the small error in the Coulomb energy (ΔE_J), this error increases when propagated to the total energy. For example, while the error in the Coulomb energy is less than 0.01 mhartree and 0.006 mhartree for *B16* and *B20*, respectively, the total energy error (ΔE_{tot}) increases to 30 mhartree and 0.04 mhartree. This amplification arises because the density-fitting scheme in BERTHA uses the fitted density ($\tilde{\rho}$) in the exchange-correlation term. A detailed comparison between the exact and density-fitted total energy expressions [Eqs. S1 and S2] shows that the primary source of error lies in the evaluation of the exchange-correlation energy functional $(E^{(l)}_{XC}[\rho(\mathbf{r})] - E^{(l)}_{XC}[\tilde{\rho}(\mathbf{r})])$:

$$E_{\text{tot}} = \sum_i \varepsilon_i - E_H^{(l)}[\rho(\mathbf{r})] + E_{XC}^{(l)}[\rho(\mathbf{r})] - \int v_{XC}^{(l)}[\rho(\mathbf{r})]\rho(\mathbf{r})\, d\mathbf{r}, \tag{S1}$$

$$\tilde{E}_{\text{tot}} = \sum_i \varepsilon_i - E_H^{(l)}[\tilde{\rho}(\mathbf{r})] + E_{XC}^{(l)}[\tilde{\rho}(\mathbf{r})] - \int v_{XC}^{(l)}[\tilde{\rho}(\mathbf{r})]\tilde{\rho}(\mathbf{r})\, d\mathbf{r}. \tag{S2}$$

By imposing a stationary condition on the density matrix $D_{\mu\nu}^{TT}$, the total density matrix obtained at the end of the SCF procedure closely reflects the true density. Thus, a simple correction scheme can be developed to improve the accuracy of the total energy. Adding the term $E_{XC}^{(l)}[\rho(\mathbf{r})] - E_{XC}^{(l)}[\tilde{\rho}(\mathbf{r})]$ to Eq. S2 significantly reduces the total energy error to the same order of magnitude as ΔE_J. This is illustrated in Table S1 (values in parentheses).

This clearly shows that it is essentially equivalent to specify the errors only for the Coulomb energies (ΔE_J) as a parameter for the accuracy of the density fitting procedure as an indicator of the quality of the auxiliary basis set for the density fitting. We can confidently say that an error in the Coulomb energy between 10^{-4} and 10^{-6} hartree, is our accuracy target for an accurate auxiliary basis sets generator in the present work.

3 Exchange-correlation term

In our framework, an exchange correlation term can be expressed as:

$$\tilde{K}_{\mu\nu}^{TT} = \frac{\partial E_{xc}[\tilde{\rho}]}{\partial D_{\mu\nu}^{TT}} = \int \frac{\delta E_{xc}[\tilde{\rho}]}{\delta \tilde{\rho}(\mathbf{r})} \frac{\partial \tilde{\rho}(\mathbf{r})}{\partial D_{\mu\nu}^{TT}} d\mathbf{r}, \tag{S3}$$

where the functional derivative gives the exchange-correlation potential:

$$\frac{\delta E_{xc}[\tilde{\rho}]}{\delta \tilde{\rho}(\mathbf{r})} = v_{xc}[\tilde{\rho}](\mathbf{r}). \tag{S4}$$

Using the fitted density,

$$\frac{\partial \tilde{\rho}(\mathbf{r})}{\partial D_{\mu\nu}^{TT}} = \sum_t \frac{\partial d_t}{\partial D_{\mu\nu}^{TT}} f_t(\mathbf{r}) = \sum_{st} A_{st}^{-1} I_{t,\mu\nu}^{TT} f_s(\mathbf{r}). \tag{S5}$$

Substituting into Eq. S3 and integrating:

$$\tilde{K}_{\mu\nu}^{TT} = \sum_{st} A_{st}^{-1} w_s I_{t,\mu\nu}^{TT} = \sum_t z_t I_{t,\mu\nu}^{TT}, \tag{S6}$$

where z_t satisfies:

$$\mathbf{A}\,\mathbf{z} = \mathbf{w}, \tag{S7}$$

and

$$w_s = \langle v_{xc}[\tilde{\rho}] | f_s \rangle \,. \tag{S8}$$

4 Supplementary benchmark tables

Here we provide the mean error on Coulomb energies for each shell of the dataset with each fitting set we have generated, both not normalized and normalized per number of electrons

Table S2: MCEE (Mean error on Coulomb energy) in a.u. and deviations for different **ABS**.

Shell	GEN-n2-v1 MCEE ± STDEV	GEN-n2-v2 MCEE ± STDEV	GEN-n3-v1 MCEE ± STDEV	GEN-n3-v2 MCEE ± STDEV
1s	$4.25 \times 10^{-5} \pm 0.0$	$1.33 \times 10^{-5} \pm 0.0$	$3.25 \times 10^{-6} \pm 0.0$	$5.36 \times 10^{-7} \pm 0.0$
2s	$1.13 \times 10^{-4} \pm 1.48 \times 10^{-4}$	$3.4 \times 10^{-5} \pm 4.53 \times 10^{-5}$	$6.74 \times 10^{-5} \pm 1.23 \times 10^{-4}$	$3.73 \times 10^{-6} \pm 3.98 \times 10^{-6}$
2p	$8.25 \times 10^{-5} \pm 5.19 \times 10^{-5}$	$3.0 \times 10^{-5} \pm 1.86 \times 10^{-5}$	$1.67 \times 10^{-4} \pm 1.52 \times 10^{-4}$	$1.26 \times 10^{-5} \pm 1.27 \times 10^{-5}$
3s	$1.69 \times 10^{-4} \pm 1.29 \times 10^{-4}$	$3.78 \times 10^{-5} \pm 2.86 \times 10^{-5}$	$2.06 \times 10^{-5} \pm 4.21 \times 10^{-5}$	$2.49 \times 10^{-6} \pm 2.74 \times 10^{-6}$
3p	$2.6 \times 10^{-4} \pm 3.24 \times 10^{-4}$	$1.2 \times 10^{-4} \pm 1.06 \times 10^{-4}$	$6.79 \times 10^{-5} \pm 6.94 \times 10^{-5}$	$1.44 \times 10^{-5} \pm 1.33 \times 10^{-5}$
3d	$1.85 \times 10^{-3} \pm 3.06 \times 10^{-3}$	$7.77 \times 10^{-5} \pm 6.56 \times 10^{-5}$	$8.9 \times 10^{-4} \pm 1.68 \times 10^{-3}$	$8.05 \times 10^{-6} \pm 8.83 \times 10^{-6}$
4s	$4.35 \times 10^{-4} \pm 4.23 \times 10^{-4}$	$8.29 \times 10^{-5} \pm 4.19 \times 10^{-5}$	$2.06 \times 10^{-5} \pm 1.15 \times 10^{-5}$	$3.83 \times 10^{-6} \pm 2.19 \times 10^{-6}$
4p	$1.6 \times 10^{-3} \pm 1.9 \times 10^{-3}$	$1.54 \times 10^{-4} \pm 2.11 \times 10^{-4}$	$1.5 \times 10^{-4} \pm 2.04 \times 10^{-4}$	$1.56 \times 10^{-5} \pm 2.32 \times 10^{-5}$
4d	$7.41 \times 10^{-4} \pm 3.72 \times 10^{-4}$	$8.4 \times 10^{-5} \pm 6.29 \times 10^{-5}$	$4.19 \times 10^{-4} \pm 8.19 \times 10^{-4}$	$1.08 \times 10^{-5} \pm 8.2 \times 10^{-6}$
4f	$3.59 \times 10^{-3} \pm 2.75 \times 10^{-3}$	$1.56 \times 10^{-4} \pm 2.1 \times 10^{-4}$	$2.15 \times 10^{-4} \pm 1.4 \times 10^{-4}$	$6.08 \times 10^{-5} \pm 9.23 \times 10^{-5}$
5s	$6.32 \times 10^{-4} \pm 5.36 \times 10^{-5}$	$2.63 \times 10^{-5} \pm 1.313 \times 10^{-5}$	$3.41 \times 10^{-5} \pm 2.23 \times 10^{-5}$	$2.27 \times 10^{-6} \pm 1.33 \times 10^{-6}$
5p	$2 \times 10^{-3} \pm 5.74 \times 10^{-4}$	$8.86 \times 10^{-5} \pm 9.16 \times 10^{-5}$	$7.98 \times 10^{-5} \pm 7.37 \times 10^{-5}$	$7.55 \times 10^{-6} \pm 9.01 \times 10^{-6}$
5d	$3.39 \times 10^{-4} \pm 1.48 \times 10^{-4}$	$7.4 \times 10^{-5} \pm 4.98 \times 10^{-5}$	$5.98 \times 10^{-5} \pm 4.22 \times 10^{-5}$	$8.82 \times 10^{-6} \pm 5.86 \times 10^{-6}$
5f	$9.56 \times 10^{-4} \pm 0.0$	$3.23 \times 10^{-4} \pm 0.0$	$1.98 \times 10^{-4} \pm 0.0$	$6.26 \times 10^{-5} \pm 0.0$
6s	$2.23 \times 10^{-3} \pm 1.22 \times 10^{-3}$	$2.52 \times 10^{-5} \pm 1.3 \times 10^{-5}$	$7.01 \times 10^{-5} \pm 4.16 \times 10^{-5}$	$5.67 \times 10^{-6} \pm 3.38 \times 10^{-6}$
6p	$5.42 \times 10^{-4} \pm 5.15 \times 10^{-4}$	$8.47 \times 10^{-5} \pm 9.24 \times 10^{-5}$	$5.87 \times 10^{-5} \pm 9.04 \times 10^{-5}$	$7.22 \times 10^{-6} \pm 9.01 \times 10^{-6}$
6d	$2.29 \times 10^{-4} \pm 9.81 \times 10^{-5}$	$5.28 \times 10^{-5} \pm 2.42 \times 10^{-5}$	$1.76 \times 10^{-4} \pm 9.6 \times 10^{-5}$	$3.94 \times 10^{-5} \pm 4.46 \times 10^{-5}$
7s	$2.42 \times 10^{-4} \pm 4.69 \times 10^{-5}$	$1.31 \times 10^{-5} \pm 4.75 \times 10^{-6}$	$4.61 \times 10^{-5} \pm 2.55 \times 10^{-5}$	$1.72 \times 10^{-5} \pm 1.63 \times 10^{-5}$
7p	$1.87 \times 10^{-4} \pm 4.61 \times 10^{-5}$	$1.79 \times 10^{-5} \pm 7.62 \times 10^{-6}$	$2.64 \times 10^{-5} \pm 1.19 \times 10^{-5}$	$2.60 \times 10^{-6} \pm 1.1 \times 10^{-6}$

Table S3: MCEE (Mean error on Coulomb energy) per electron in a.u. and deviations for different **ABS**.

Shell	GEN-n2-v1 MCEE ± STDEV	GEN-n2-v2 MCEE ± STDEV	GEN-n3-v1 MCEE ± STDEV	GEN-n3-v2 MCEE ± STDEV
1s	$3.04 \times 10^{-6} \pm 0.0$	$9.48 \times 10^{-7} \pm 0.0$	$2.32 \times 10^{-7} \pm 0.0$	$3.83 \times 10^{-8} \pm 0.0$
2s	$4.29 \times 10^{-6} \pm 4.69 \times 10^{-6}$	$1.2 \times 10^{-7} \pm 7.06 \times 10^{-7}$	$2.44 \times 10^{-6} \pm 4.62 \times 10^{-6}$	$1.38 \times 10^{-7} \pm 8.44 \times 10^{-8}$
2p	$4.16 \times 10^{-6} \pm 1.97 \times 10^{-6}$	$1.45 \times 10^{-6} \pm 5.55 \times 10^{-7}$	$7.41 \times 10^{-6} \pm 4.66 \times 10^{-6}$	$5.6 \times 10^{-7} \pm 4.57 \times 10^{-7}$
3s	$4.88 \times 10^{-6} \pm 2.05 \times 10^{-6}$	$1.1 \times 10^{-6} \pm 5.86 \times 10^{-7}$	$4.93 \times 10^{-7} \pm 8.55 \times 10^{-7}$	$6.4 \times 10^{-8} \pm 5.34 \times 10^{-8}$
3p	$5.7 \times 10^{-6} \pm 4.57 \times 10^{-6}$	$2.79 \times 10^{-6} \pm 1.53 \times 10^{-6}$	$1.55 \times 10^{-6} \pm 1.3 \times 10^{-6}$	$3.28 \times 10^{-7} \pm 2.52 \times 10^{-7}$
3d	$3.36 \times 10^{-5} \pm 5.68 \times 10^{-5}$	$1.3 \times 10^{-6} \pm 8.12 \times 10^{-7}$	$1.63 \times 10^{-5} \pm 3.16 \times 10^{-5}$	$1.39 \times 10^{-7} \pm 1.18 \times 10^{-7}$
4s	$8.56 \times 10^{-6} \pm 5.44 \times 10^{-6}$	$1.94 \times 10^{-6} \pm 6.85 \times 10^{-7}$	$4.5 \times 10^{-7} \pm 1.1 \times 10^{-7}$	$8.55 \times 10^{-8} \pm 3.02 \times 10^{-8}$
4p	$1.78 \times 10^{-5} \pm 8.36 \times 10^{-6}$	$1.43 \times 10^{-6} \pm 1.16 \times 10^{-6}$	$1.47 \times 10^{-6} \pm 1.03 \times 10^{-6}$	$1.47 \times 10^{-7} \pm 1.19 \times 10^{-7}$
4d	$1.07 \times 10^{-5} \pm 6.89 \times 10^{-6}$	$1.19 \times 10^{-6} \pm 1.08 \times 10^{-6}$	$6.01 \times 10^{-6} \pm 1.24 \times 10^{-5}$	$1.44 \times 10^{-7} \pm 1.1 \times 10^{-7}$
4f	$2.68 \times 10^{-5} \pm 1.57 \times 10^{-5}$	$1.78 \times 10^{-6} \pm 2.55 \times 10^{-6}$	$2.16 \times 10^{-6} \pm 1.67 \times 10^{-6}$	$6.99 \times 10^{-7} \pm 1.11 \times 10^{-6}$
5s	$1.38 \times 10^{-5} \pm 2.1 \times 10^{-6}$	$5.49 \times 10^{-7} \pm 2.27 \times 10^{-7}$	$7.57 \times 10^{-7} \pm 5.73 \times 10^{-7}$	$4.64 \times 10^{-8} \pm 2.38 \times 10^{-8}$
5p	$2.92 \times 10^{-5} \pm 6.6 \times 10^{-5}$	$1.08 \times 10^{-6} \pm 6.62 \times 10^{-7}$	$1 \times 10^{-6} \pm 5.15 \times 10^{-7}$	$8.93 \times 10^{-8} \pm 8.44 \times 10^{-8}$
5d	$3.07 \times 10^{-6} \pm 1.23 \times 10^{-6}$	$6.42 \times 10^{-7} \pm 3.38 \times 10^{-7}$	$5.48 \times 10^{-7} \pm 3.49 \times 10^{-7}$	$8.16 \times 10^{-8} \pm 5.66 \times 10^{-8}$
5f	$6.55 \times 10^{-6} \pm 0.0$	$2.21 \times 10^{-6} \pm 0.0$	$1.36 \times 10^{-6} \pm 0.0$	$4.29 \times 10^{-7} \pm 0.0$
6s	$3.39 \times 10^{-5} \pm 1.85 \times 10^{-5}$	$3.81 \times 10^{-7} \pm 1.67 \times 10^{-7}$	$1.11 \times 10^{-6} \pm 7.38 \times 10^{-7}$	$8.62 \times 10^{-8} \pm 5.18 \times 10^{-8}$
6p	$3.89 \times 10^{-6} \pm 1.63 \times 10^{-6}$	$6.09 \times 10^{-7} \pm 4.59 \times 10^{-7}$	$3.45 \times 10^{-7} \pm 3.27 \times 10^{-7}$	$4.64 \times 10^{-8} \pm 4.44 \times 10^{-8}$
6d	$1.89 \times 10^{-6} \pm 8.75 \times 10^{-7}$	$4.27 \times 10^{-7} \pm 1.76 \times 10^{-7}$	$1.4 \times 10^{-6} \pm 6.78 \times 10^{-7}$	$3.11 \times 10^{-7} \pm 3.35 \times 10^{-7}$
7s	$2.71 \times 10^{-6} \pm 4.97 \times 10^{-7}$	$1.47 \times 10^{-7} \pm 5.17 \times 10^{-8}$	$5.15 \times 10^{-7} \pm 2.81 \times 10^{-7}$	$1.91 \times 10^{-7} \pm 1.81 \times 10^{-7}$
7p	$1.55 \times 10^{-6} \pm 3.62 \times 10^{-7}$	$1.46 \times 10^{-7} \pm 5.55 \times 10^{-8}$	$2.2 \times 10^{-7} \pm 1.02 \times 10^{-7}$	$2.14 \times 10^{-8} \pm 8.12 \times 10^{-9}$

Table S4: MCEE (Mean error on Coulomb energy) in a.u. and deviations for the GEN-n3-v1 ABS generated from the dyall.vtz principal basis set

**Shell**	**GEN-n3-v1**<br>**MCEE ± STDEV**
1s	2.56×10^{-7} ±0.0
2s	$1.08 \times 10^{-5} \pm 1.88 \times 10^{-5}$
2p	$2.56 \times 10^{-5} \pm 9.23 \times 10^{-5}$
3s	$1.78 \times 10^{-5} \pm 3.22 \times 10^{-5}$
3p	$1.96 \times 10^{-4} \pm 3.27 \times 10^{-4}$
3d	$1.2 \times 10^{-4} \pm 3.45 \times 10^{-4}$
4s	$2.88 \times 10^{-5} \pm 4.29 \times 10^{-5}$
4p	$6.32 \times 10^{-5} \pm 1.6 \times 10^{-4}$
4d	$1 \times 10^{-4} \pm 2.05 \times 10^{-4}$
4f	$9,89 \times 10^{-5} \pm 6.97 \times 10^{-5}$
5s	$1.05 \times 10^{-4} \pm 7.09 \times 10^{-5}$
5p	$6.98 \times 10^{-5} \pm 1.08 \times 10^{-4}$
5d	$1.73 \times 10^{-5} \pm 2.74 \times 10^{-5}$
6s	$6.33 \times 10^{-5} \pm 6.03 \times 10^{-5}$
6p	$3.89 \times 10^{-5} \pm 9.04 \times 10^{-5}$
6d	$3.29 \times 10^{-5} \pm 4.28 \times 10^{-5}$
7s	$8.55 \times 10^{-6} \pm 3.15 \times 10^{-6}$
7p	$1.96 \times 10^{-6} \pm 1.45 \times 10^{-6}$

References

(1) Belpassi, L.; Tarantelli, F.; Sgamellotti, A.; Quiney, H. M. Electron density fitting for the Coulomb problem in relativistic density-functional theory. **2006**, *124*, 124104.

(2) Quiney, H.; Belanzoni, P.; Sgamellotti, A. Evaluation of the Coulomb energy in relativistic self-consistent-field theory. *Theoretical Chemistry Accounts* **2002**, *108*, 113–123.
